# Room-Temperature Interconversion Between Ultrathin CdTe Magic-Size Nanowires Induced by Ligand Shell Dynamics

**DOI:** 10.1021/acs.jpcc.2c04113

**Published:** 2022-08-31

**Authors:** Serena Busatto, Claudia Spallacci, Johannes D. Meeldijk, Stuart Howes, Celso de Mello Donega

**Affiliations:** † Condensed Matter and Interfaces, Debye Institute for Nanomaterials Science, Utrecht University, 3508 TA Utrecht, The Netherlands; ‡ Materials Chemistry and Catalysis, Debye Institute for Nanomaterials Science, Utrecht University, 3508 TA Utrecht, The Netherlands; § Structural Biochemistry, Bijvoet Centre for Biomolecular Research, Utrecht University, Padualaan 8, 3584 CH Utrecht, The Netherlands

## Abstract

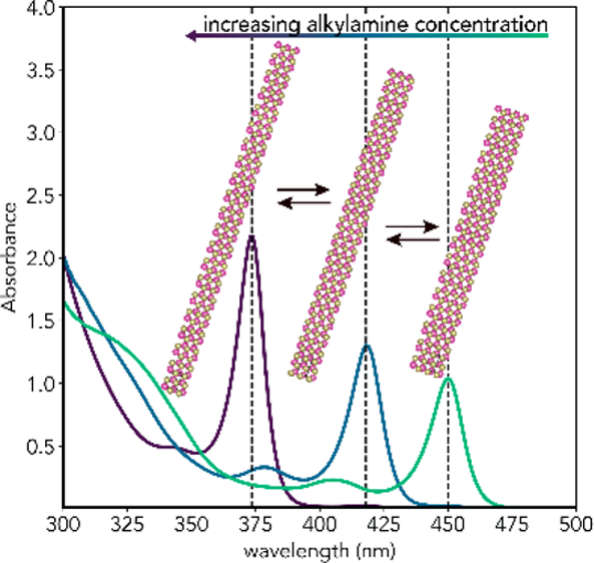

The formation mechanisms of colloidal magic-size semiconductor nanostructures have remained obscure. Herein, we report the room temperature synthesis of three species of ultrathin CdTe magic-size nanowires (MSNWs) with diameters of 0.7 ± 0.1 nm, 0.9 ± 0.2 nm, and 1.1 ± 0.2 nm, and lowest energy exciton transitions at 373, 418, and 450 nm, respectively. The MSNWs are obtained from Cd(oleate)_2_ and TOP-Te, provided diphenylphosphine and a primary alkylamine (RNH_2_) are present at sufficiently high concentrations, and exhibit sequential, discontinuous growth. The population of each MSNW species is entirely determined by the RNH_2_ concentration [RNH_2_] so that single species are only obtained at specific concentrations, while mixtures are obtained at concentrations intermediate between the specific ones. Moreover, the MSNWs remain responsive to [RNH_2_], interconverting from thinner to thicker upon [RNH_2_] decrease and from thicker to thinner upon [RNH_2_] increase. Our results allow us to propose a mechanism for the formation and interconversion of CdTe MSNWs and demonstrate that primary alkylamines play crucial roles in all four elementary kinetic steps (viz., monomer formation, nucleation, growth in length, and interconversion between species), thus being the decisive element in the creation of a reaction pathway that leads exclusively to CdTe MSNWs. The insights provided by our work thus contribute toward unravelling the mechanisms behind the formation of shape-controlled and atomically precise magic-size semiconductor nanostructures.

## 1 Introduction

The optoelectronic properties of colloidal semiconductor nanocrystals (NCs) can be tailored by controlling their size, shape, and composition.^1−3^ Moreover, these nanomaterials are coated with ligands and therefore can easily be processed from solution. These characteristics have turned them into promising materials for a myriad of applications.^1,4−8^ However, the size and shape polydispersity of ensembles of most NC compositions remain relatively large, severely limiting their utilization. This has motivated worldwide research efforts into the development of synthesis protocols to narrow the size and shape dispersion of colloidal NCs and prompted many groups to explore possible routes toward atomically precise synthesis.^9−12^

It has been recently pointed out that, although atomically precise synthesis remains elusive for most colloidal semiconductor NCs, it is already within reach for a class of nanomaterials referred to as “magic-size nanostructures” (MSNSs).^13^ Semiconductor MSNSs are in the strong quantum confinement regime and can be zero-dimensional (0D, magic-size clusters and magic-size quantum dots), one-dimensional (1D, ultrathin nanowires), two-dimensional (2D, ultrathin nanosheets), or quasi-2D (weak confinement occurs also in the lateral directions, e.g., ultrathin nanoribbons and nanoplatelets).^13^ The term “magic-size” is used to denote that at least one critical dimension (the confinement dimension, i.e., diameter or thickness) is atomically precise and changes only in discrete steps from one species to the next (i.e., growth is discontinuous or quantized).^13^ The idea that discontinuous growth is restricted to the cluster or ultrathin size regime (≤2 nm) has been recently questioned since discrete growth of CdSe nanostructures was observed up to ∼3.5 nm,^9,11,14^ which is well into the size range of colloidal quantum dots (QDs). Moreover, transformation and interconversion of MSNSs in response to external stimuli have been reported by several groups.^15−19^

These observations raise the exciting possibility that the underlying mechanism responsible for the enhanced stability and stepwise growth that characterizes MSNSs may hold the key to extend and generalize atomically precise synthesis. Nonetheless, the formation and interconversion mechanisms of MSNSs remain poorly understood, despite recent advances identifying correlations between the formation of magic-size clusters and 0D, 1D, and 2D MSNSs.^12,13,20,21^ The insights provided by these studies suggest that the driving force behind the formation of MSNSs emerges from the complexity of a dynamic system running under reaction control.^13^ However, the intricate framework of synergistic and antagonistic interactions that occur between multiple and dynamic variables as the reaction progresses has yet to be elucidated. In particular, the exact role of ligands and their relevance remain obscure.^13^ Given the very high surface to volume ratio of MSNSs, one would expect ligands to play a vital role in their formation since ligands have been shown to have a critical impact on colloidal semiconductor NCs, affecting their nucleation and growth kinetics, faceting, shape, and composition, self-organization behavior, and optoelectronic properties.^3,22−29^

Here, we unravel the crucial roles played by ligands (primary alkylamines) on the formation and interconversion of MSNSs by investigating three species of micrometer-long ultrathin CdTe magic-size nanowires (MSNWs) that form at room temperature from cadmium oleate and trioctylphosphine-telluride, provided diphenylphosphine and a primary alkylamine (RNH_2_) are present. The paper is organized as follows. We first provide a general characterization of the CdTe MSNWs by optical absorption spectroscopy, transmission electron microscopy (TEM), and cryo-TEM (section 3.1). This analysis shows that the CdTe MSNWs have diameters of 0.7 ± 0.1 nm, 0.9 ± 0.2 nm, and 1.1 ± 0.2 nm (3, 4, and 5 atomic monolayers, respectively), and lowest energy exciton transitions at 373, 418, and 450 nm, respectively. We refer to them hereafter according to the spectral position of their lowest energy absorption transition: NW-373, NW-418, and NW-450, respectively. We then analyze the impact of alkylamines on the formation of the MSNWs (section 3.2) and show that they only form if primary alkylamines (regardless of their chain length) are present above a critical concentration (≥0.1 M). The population of each MSNW species is determined entirely by the RNH_2_ concentration [RNH_2_] so that single-species are only obtained at specific concentrations (viz., 0.29, 0.87, and ≥2.0 M, for NW-450, NW-418, and NW-373, respectively), while mixtures of two different species are obtained at concentrations intermediate between the specific ones. At [RNH_2_] lower than 2 M, formation of MSNWs is accompanied by quantized growth, which is equivalent to interconversion between MSNW species. Intriguingly, the MSNWs remain responsive to [RNH_2_], interconverting from thinner to thicker upon [RNH_2_] reduction and from thicker to thinner upon increase of [RNH_2_]. The monomer formation mechanism is discussed in section 3.3, while a formation mechanism for the ultrathin CdTe MSNWs is proposed in section 3.4. The dynamics of the formation and interconversion of the CdTe MSNWs are analyzed in detail in section 3.5. Finally, in section 3.6. we combine the experimental findings and insights provided by the preceding sections into a comprehensive mechanism for the formation of and reversible interconversion between CdTe MSNWs. Our analysis demonstrates that primary alkylamines are essential in all four elementary kinetic steps of the formation of the MSNWs (viz., monomer formation, nucleation, growth in length, and interconversion between species). Most importantly, they play a decisive role in directing the reaction toward a pathway that leads exclusively to CdTe MSNWs. Notably, our results show that primary alkylamines exert this role through a dynamic 1D-directive effect driven by the concomitant formation of a fully packed RNH_2_ monolayer at the surface of the growing MSNW, allowing us to rule out templating and oriented attachment mechanisms.^21^ The responsiveness of the MSNWs to the primary alkylamine concentration originates from the same driving force, given that RNH_2_ monolayers on the surface of CdTe NCs are known to be very dynamic.^3^ The insights provided by our work lead to a deeper understanding of the formation of ultrathin colloidal CdTe magic-size nanowires and thus contribute toward unravelling the mechanisms behind the formation of shape-controlled and atomically precise semiconductor nanostructures.

## 2 Experimental Methods

### 2.1 Materials

The chemicals were purchased from Sigma-Aldrich unless otherwise stated: Toluene (99.8%, anhydrous, Alfa Aesar), 1-octadecene (ODE, technical grade, 90%), butylamine (99.5%), amylamine (99%), hexylamine (99%), heptylamine (99%), octylamine (99%), di-*n*-octylamine (97%), tri-*n*-octylamine (98%), dodecylamine (DDA, 98%), octadecylamine (technical grade, 90%), oleylamine (OLA, ≥ 98%), lithium triethylborohydride (LiEt_3_BH, Super-Hydride, 1 M in THF). cadmium oxide (CdO, 99.99%), tellurium powder (99.997%, −30 mesh), oleic acid (technical grade, 90%), trioctylphosphine (TOP, technical grade, 90%), and diphenylphosphine (DPP, 98%). All chemicals were used as received except for ODE and OLA, which were degassed prior to use (∼1 mbar at 100 °C for 4 h).

### 2.2 Cadmium and Tellurium Precursor Solutions

0.1 M Cd(oleate)_2_ in ODE was prepared by heating 1.284 g (0.01 mol) of CdO and 11.299 g (0.04 mol) of oleic acid in 100 mL of ODE at 220 °C in a Schlenk line under N_2_ for 2 h until all the CdO was dissolved. The clear solution was cooled to 100 °C and degassed under a vacuum for 2 h. A 0.05 M solution of TOP-Te in TOP was obtained by dissolving 31.9 mg (0.25 mmol) of Te powder in 5 mL of TOP at 50 °C for 1 h inside a N_2_-filled glovebox (≤2 ppm of O_2_ and <1 ppm of H_2_O).

### 2.3 Synthesis of Colloidal CdTe Magic-Size Nanowires (MSNWs)

Colloidal suspensions of CdTe MSNWs were prepared by adding 100 μL of 0.1 M Cd-oleate in ODE, 100 μL of 0.05 M TOP-Te in TOP, and 10 μL of DPP to 2.79 mL of a solution of variable amounts of DDA (0 to 2 M) in anhydrous toluene (total volume: 3.000 mL) and allowing the mixture to react at room temperature for variable amounts of time (1.5 min to 4 days). Neat DDA was also used as the reaction solvent. Depending on the DDA concentration, different CdTe MSNW species were obtained, either as single or mixed species. The effect of using different alkylamines (primary, secondary, or tertiary) and different DPP concentrations was also investigated. The progress of the reaction was followed by absorption spectroscopy. Selected samples were also analyzed by transmission electron microscopy. All reactions were carried out in a N_2_-filled glovebox (≤2 ppm of O_2_ and <1 ppm of H_2_O).

### 2.4 Absorption Spectroscopy

The synthesis of the CdTe MSNWs and sample preparation were carried out in the same N_2_-filled glovebox. Samples for optical measurements were prepared by diluting either 150 or 250 μL aliquots of the reaction mixture to 3.000 mL with anhydrous toluene in 10 mm optical path length sealable quartz cuvettes. Measurements were also carried out using solutions of DDA in toluene instead of neat toluene as the solvent. Absorption spectra were recorded on a double-beam PerkinElmer Lambda 950 UV/vis/NIR spectrophotometer (250–700 nm), immediately (≤30 s) after dilution of the samples. The temporal evolution of the absorption spectra of the reaction mixture was also followed without any dilution using 1 mm optical path length sealable quartz cuvettes.

### 2.5 Conventional Transmission Electron Microscopy (TEM)

Conventional TEM images and high angle annular dark field scanning TEM (HAADF-STEM) images were acquired on a Thermo Fisher Scientific Talos F200X microscope, operating at 200 kV. Samples for TEM imaging were prepared by diluting the CdTe reaction mixture in toluene and drop-casting the solutions shortly after dilution onto carbon-coated 200 mesh copper TEM grids.

### 2.6 Cryo-Transmission Electron Microscopy (Cryo-TEM)

Cryo-TEM images were acquired from frozen films of the diluted solutions on holey carbon–copper grids. The films were prepared by placing a droplet of solution on the grid and using a Vitrobot (Thermo Fisher Scientific) with toluene-soaked filter papers inside the environmental chamber to automatically blot the grid and plunge into liquid nitrogen. The grids were either transferred to a FEI Tecnai20F microscope operating at 120 kV using a Gatan 626 cryo-transfer holder or clipped into autogrid cartridges and transferred to a Thermo Fisher Scientific Talos Arctica microscope operating at 200 kV and imaged in brightfield mode using a K2 detector (Gatan). For low e-dose conditions, the total dose was kept below a maximum of 10 electrons per Å^2^.

## 3 Results and Discussion

### 3.1 Room-Temperature Synthesis of Ultrathin Colloidal CdTe Magic-Size Nanowires

**Figure 1:**
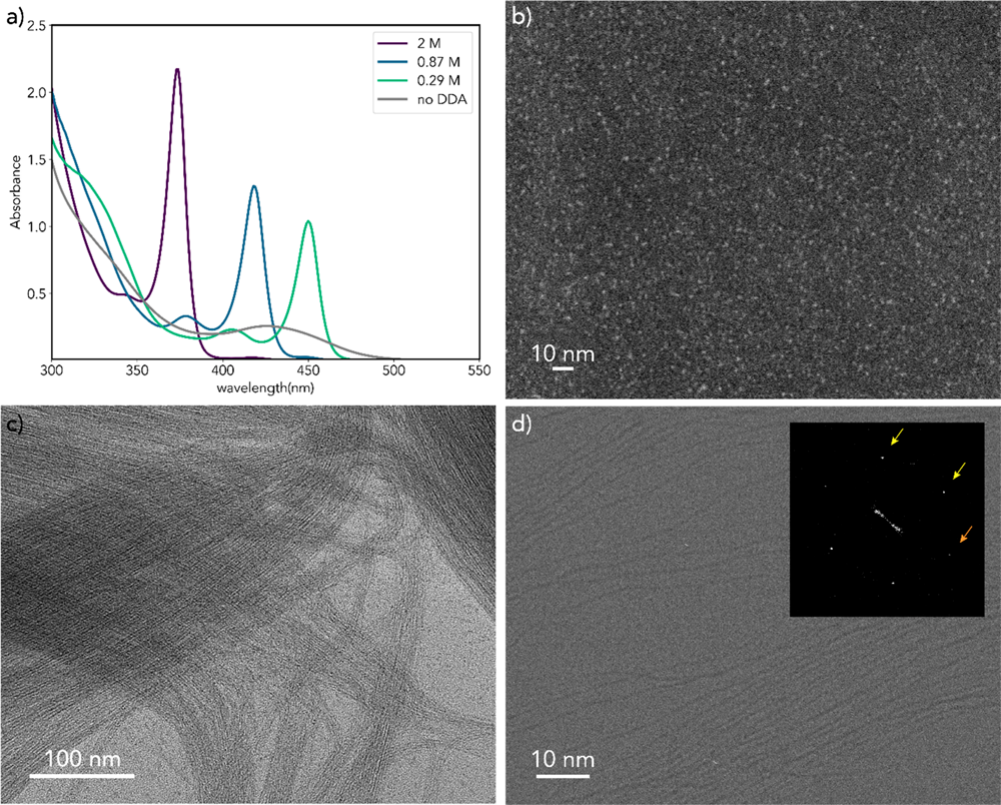
(a) Absorption spectra of the CdTe products obtained after 24 h of reaction for different concentrations of dodecylamine (DDA). All other reaction variables and the dilution factor of the reaction mixture for the absorption spectra measurements are the same. (b) High angle annular dark field scanning TEM image of the CdTe product obtained without DDA (gray curve in panel a). (c, d) Conventional (c) and low electron-dose cryo-TEM (d) images of the CdTe product obtained with 0.29 M DDA (green curve in panel a). The inset in panel (d) shows the fast Fourier transform (FFT) pattern (the yellow and orange arrows indicate interplanar spacings of 3.7 and 4.1 Å, respectively). Additional conventional and cryo-TEM images of this sample using higher e-doses or exposure times are shown in the Supporting Information, Figures S2 and S3. Low e-dose cryo-TEM images of the products obtained with 0.87 and 2.04 M DDA (blue and purple curves in panel a) are shown in Figure S1.

The absorption spectra and transmission electron microscopy (TEM) images of the CdTe products obtained after 24 h of reaction at room temperature under different concentrations of dodecylamine (DDA) are shown in Figure 1. In the absence of DDA, the absorption spectrum is characterized by broad features with the lowest energy absorption peak centered at 426 nm (2.91 eV) and a full width at half-maximum (fwhm) of 300 meV (Figure 1a). These absorption transitions can be ascribed to an ensemble of CdTe QDs with an average diameter of 1.8 ± 0.2 nm, as demonstrated by high angle annular dark field scanning TEM (HAADF-STEM) measurements (Figure 1b). We note that the average diameter obtained from the HAADF-STEM images is in good agreement with that estimated from the peak position of the lowest energy exciton transition using previously published sizing curves (viz., 2.1 ± 0.2 nm).^30^ In striking contrast, the absorption spectra of the CdTe products obtained in the presence of DDA are characterized by very narrow (fwhm = 128 ± 7 meV) lowest energy absorption peaks at spectral positions that are dictated by the DDA concentration: 373 nm (3.32 eV) for 2 M, 418 nm (2.97 eV) for 0.87 M, and 450 nm (2.76 eV) for 0.29 M (Figure 1a). Similar absorption spectra have been previously reported by Yu and co-workers for CdTe nanostructures obtained by a two-step procedure in which aliquots of solutions prepared by heating Cd(oleate)_2_ and TOP-Te at temperatures between 60 and 130 °C for 10–30 min were diluted in solutions of octylamine (OTA) in toluene at room temperature.^17,18^ Depending on the OTA concentration and time after dilution, CdTe species exhibiting narrow lowest energy absorption peaks at 371 nm ([OTA] = 2 M, immediately after dilution), 417 nm ([OTA] = 1 M, 60 min after dilution) or 448 nm ([OTA] = 0.4 M, 120 min after dilution) were observed and attributed to three different species of CdTe magic-sized clusters.^17,18^ This assignment, however, was based solely on the absorption spectra.^17,18^ In contrast, TEM and cryo-TEM measurements unambiguously establish that the CdTe species responsible for the narrow absorption peaks observed in our work (Figure 1a) are in fact micrometer long ultrathin nanowires with diameters that depend on the DDA concentration (viz., 0.7 ± 0.1 nm for 2 M, 0.9 ± 0.2 nm for 0.87 M, and 1.1 ± 0.2 nm for 0.29 M) (Figures 1c,d, and S1). Importantly, the fact that TEM and cryo-TEM yield the same results excludes the possibility that the nanowires are formed ex-situ on the TEM grids and demonstrates that they are already present in the reaction solution.

As will be discussed in the following sections, these three ultrathin CdTe nanowire species are the only possible reaction products at sufficiently high alkylamine concentrations, growing sequentially during the reaction and interconverting into each other under suitable conditions. It should be noted that the relatively large standard deviation of the diameters estimated from the cryo-TEM measurements (±20%) does not accurately reflect the actual ensemble polydispersity since it can be attributed to the low contrast of the images and the limited number of nanowires analyzed. Given that these ultrathin CdTe nanowires experience extremely strong 1D quantum confinement potentials (viz., 1.2–1.76 eV, which correspond to 77–113% of the bulk bandgap),^30^ any diameter fluctuation should result in dramatic changes in the spectral position of the exciton transitions. As a result, the fwhm of the lowest energy absorption peak provides a more reliable and accurate way to assess the polydispersity and heterogeneity of nanowire ensembles.^31^ Unfortunately, sizing curves for CdTe nanowires are only available for diameters larger than 5 nm,^32^ so we used instead the sizing curve previously reported for CdTe QDs.^30^ This overestimates the diameter of the nanostructures because the confinement potential of QDs is larger than that of nanowires since in the latter case the exciton is confined in only two dimensions.^32^ Nonetheless, this should provide a reliable estimate for the diameter polydispersity. This analysis shows that lowest energy exciton transitions at 3.32, 2.97, and 2.76 eV with a fwhm of ∼130 meV (Figure 1a) can be ascribed to ensembles of CdTe QDs with average diameters of 1.65 ± 0.05 nm, 1.95 ± 0.05 nm, and 2.18 ± 0.06 nm, respectively. Although the diameters are overestimated because the nanostructures are in fact 1D nanowires rather than 0D QDs, the standard deviations (∼0.5 Å, which is smaller than the diameters of both Cd^2+^ and Te^2–^, 1.56 and 4.42 Å, respectively) can be used to assess the diameter polydispersity, implying that the ultrathin CdTe nanowires investigated in our work have atomically precise diameters that increase in discrete steps from one species to the next. We thus propose that they can be categorized as magic-size nanowires (MSNWs).^13^ For convenience, we will hereafter refer to them according to the spectral position of their lowest energy absorption transitions: NW-373, NW-418, and NW-450 for the MSNWs with the lowest energy exciton transition at 373, 418, and 450 nm, respectively.

Owing to their extremely narrow diameters, these MSNWs are extremely sensitive to the electron beam. As a result, they quickly undergo electron-beam induced structural reconstruction, thereby appearing as segmented nanowires or strings of nanoclusters upon irradiation with high electron doses (either upon higher magnification or prolonged exposure) (Figures S2 and S3). This pronounced e-beam sensitivity has unfortunately precluded the structural analysis of the MSNWs by high-resolution TEM and STEM. Nevertheless, low electron dose cryo-TEM images unequivocally show that the CdTe MSNWs prepared in our work are not only continuous but also single-crystalline, as demonstrated by the fast Fourier transform (FFT) patterns of the images (Figures 1d and S1). The interplanar spacings extracted from the FFT patterns (viz., 4.1 and 3.7 Å for NW-450, Figure 1d, 4.1 and 3.8 Å for NW-373, Figure S1a, and 4.3 and 3.6 Å for NW-418, Figure S1b) can be assigned to, respectively, the (11̅0) and the (002) planes of wurtzite CdTe (bulk values: 3.95 and 3.75 Å, respectively).^33^ We thus propose that the ultrathin CdTe MSNWs have a wurtzite structure, with slightly expanded (∼1–4%) interplanar distances due to their extremely narrow diameters, which are comparable to the lattice parameters of bulk wurtzite CdTe (viz., *a* = *b* = 0.457 nm, *c* = 0.748 nm).^33^ Moreover, the fact that only the interplanar spacings associated with the (002) and (11̅0) planes are observed in the FFT patterns implies that the long axis of the nanowires corresponds to the *c*-axis of the wurtzite structure; i.e., the CdTe MSNWs grow in the <001> direction. This assignment is consistent with previous reports on nanowires of several II–VI semiconductors (viz., 2.5–11 nm diameter CdTe,^31,32,34^ 1.5–6 nm diameter CdSe,^35^ 2.4 nm diameter ZnSe,^36^ and 2 nm diameter (Zn,Cd)Te/CdSe heteronanowires^37^), which invariably adopt the wurtzite crystal structure with the long axis parallel to the *c*-axis. Further, recent DFT calculations have shown that ultrathin (0.99 nm diameter, 6 atomic monolayers thick) wurtzite ZnSe nanowires are sufficiently stable to form, growing in the <001> direction with lateral surfaces consisting of nonpolar facets.^38^ The diameters of the CdTe MSNWs prepared in our work (viz., 0.7 ± 0.1 nm, 0.9 ± 0.2 nm, and 1.1 ± 0.2 nm) can thus be compared to the expected thicknesses of 3, 4, and 5 atomic monolayers of wurtzite CdTe, which correspond to 0.686 nm (1.5*a*), 0.914 nm (2.0*a*), and 1.143 nm (2.5*a*), respectively (*a* = 0.457 nm).^33^

### 3.2 Impact of Alkylamines on the Formation of CdTe Magic-Size Nanowires

The results above clearly demonstrate that DDA has a crucial role in the formation of ultrathin CdTe MSNWs and exerts its influence in a concentration-dependent fashion. This critical role is not dependent on the alkyl chain length since any primary alkylamine (from butyl- to dodecylamine) has essentially the same effect (Figure S4). On the other hand, secondary and tertiary alkylamines do not yield nanowires, similarly to when amines are absent in the reaction medium (Figure S5). Possible reasons for the different impact of primary, secondary, and tertiary alkylamines will be discussed in section 3.4 below. To gain insight into the role of alkylamines in the formation of CdTe MSNWs at room temperature, we followed the evolution of the reaction over time, using DDA as a representative primary alkylamine. As pointed out in ref (13) and clearly demonstrated above (Figure 1), the use of absorption spectroscopy as the sole technique to study magic-size nanostructures is questionable because it cannot unambiguously identify the species responsible for the absorption transitions. Nevertheless, after the identity of the absorbing species has been established by complementary techniques, such as TEM and cryo-TEM, absorption spectroscopy becomes a powerful and convenient technique to follow the formation dynamics of the nanostructures in their native environment (i.e., in solution).

As shown above (Figure 1), single-species CdTe MSNWs are obtained after 24 h of reaction using specific DDA concentrations ([DDA] = 0.29, 0.87, and 2.04 M, for NW-450, NW-418, and NW-373, respectively). The temporal evolution of the absorption spectra of the CdTe products obtained for these three [DDA] shows that the first MSNW species to appear is the NW-373, regardless of [DDA] (Figure S6a–c). At [DDA] ≥ 2.0 M, NW-373 (0.7 ± 0.1 nm diameter, Figure S1a) remain as the sole MSNW species during the entire duration of the reaction, simply increasing in concentration and/or length over time, as demonstrated by the increase in absorbance of its characteristic spectral features (Figure S6c). However, at 0.87 M the characteristic absorption of NW-373 reaches a maximum and thereafter decreases over time as the intensity of the transitions associated with NW-418 (0.9 ± 0.2 nm diameter, Figure S1b) increases, until NW-418 becomes the only species present (Figure S6b). At 0.29 M, the concentration of NW-418 also starts to decrease after reaching a maximum, while that of NW-450 (1.1 ± 0.2 nm diameter, Figure 1c,d) concomitantly increases until these MSNWs become the sole species present. Interestingly, if the [DDA] intermediate to the specific ones are used, solutions containing two different MSNW species are obtained when the reaction reaches completion (Figure S6d–i). For example, 1.46 M DDA yields a mixture of NW-373 and NW-418 MSNWs (Figure S6h), while 0.58 M yields a mixture of NW-450 and NW-418 MSNWs (Figure S6d). Intriguingly, the stability of the MSNW species is directly correlated with the primary alkylamine concentration, with higher concentrations favoring narrower diameters. These observations demonstrate that the ultrathin CdTe MSNWs grow in a quantized way, jumping from one “magic”-diameter to the next until they reach the largest possible magic-diameter (or combination thereof) that is stable at a given primary alkylamine concentration. This intriguing behavior will be investigated in more detail in section 3.5 below.

**Figure 2:**
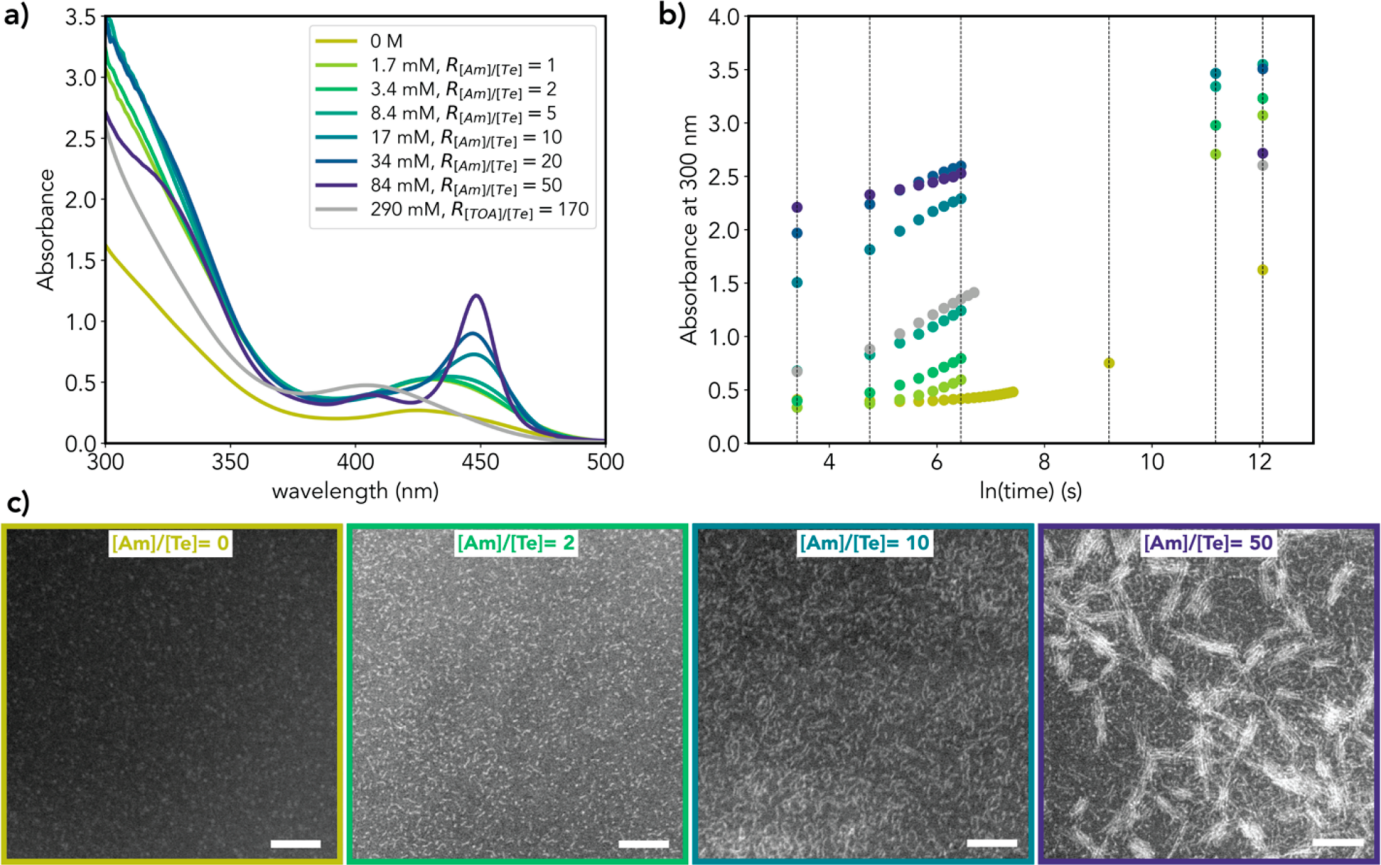
(a) Absorption spectra of the CdTe products obtained after 48 h of reaction for 0.29 M trioctylamine (TOA) and for different concentrations of dodecylamine (DDA) indicated in the legend. All other reaction variables and the dilution factor of the reaction mixture are the same ([DPP] = 33 mM). *R*_[Am]/[Te]_ gives the molar ratio between DDA and tellurium (the limiting reagent, present as TOP-Te). *R*_[TOA]/[Te]_ gives the molar ratio between TOA and tellurium. (b) Temporal evolution of the absorbance at 300 nm of the CdTe products obtained for [DDA] indicated in the legend and for 0.29 M TOA (note that time is given on a logarithmic scale, and data points span from 90 s to 48 h). The dashed lines mark 30 s, 2 min, 14 min, 3, 24, and 48 h, respectively. The absorbance values were obtained from spectra acquired in cuvettes with a 1 mm optical path using the neat reaction mixture. Color code is the same as in panel (a). (c) HAADF-STEM images of the CdTe products obtained after 48 h of reaction for selected [DDA]. The panels are labeled by the molar ratio between DDA and tellurium ([Am]/[Te]). The samples are the same used to obtain the absorption spectra shown in (a). The scale bar is 10 nm in all panels.

To establish whether there is a critical primary alkylamine concentration for the formation of CdTe MSNWs, we will address first the low concentration limit. Figure 2a presents the absorption spectra of the CdTe products obtained after 48 h of reaction for [DDA] ranging from 1.7 mM (equimolar with the amount of tellurium, which is the limiting reactant) to 84 mM. For comparison, the absorption spectra of the products obtained in the absence of amines and for 290 mM trioctylamine (TOA) are also shown. STEM images of selected samples are also shown. It is evident that very small (*d* ≈ 2 nm) prolate CdTe nanocrystals (NCs) are obtained already for [DDA] as low as 3.4 mM. These small NCs are the main reaction products for [DDA] below 10 mM and are characterized by broad absorption bands, which redshift continuously as they grow (Figure S7). This implies that they are in the continuous growth regime characteristic of colloidal semiconductor NCs,^3^ despite having dimensions typically associated with the magic-size growth regime (≤2 nm).^13^ As the [DDA] increases above 10 mM, the absorption spectra become increasing asymmetric due to the convolution of the broad absorption features characteristic of small CdTe NCs and a sharper absorption peak at ∼440 nm, attributed to the short and twisted ultrathin nanowire fragments that are observed in the STEM images coexisting with small prolate NCs. The characteristic absorption spectrum of NW-450 only becomes evident for [DDA] ≥ 84 mM, when the length of the nanowires (∼13 nm, Figure 2c) becomes sufficiently long with respect to the exciton Bohr radius of CdTe (viz., 7.3 nm)^30^ to allow them to enter the 1D quantum confinement regime. Upon increasing [DDA] beyond this critical value, long nanowires become the dominant reaction product. The short CdTe nanowires formed at [DDA] close to the critical concentration have a limited stability, as evidenced by the broadening of their absorption features upon dilution (Figure S8). We can thus conclude that primary alkylamines induce anisotropic CdTe growth even at relatively low concentrations (∼10 mM) and promote the formation of nanowires at concentrations above a critical value (0.1 M). In contrast, tertiary and secondary alkylamines yield only very small (∼2 nm diameter) NCs, even at high concentrations (Figures 2a and S5), similar to when no amines are present (Figure 2a).

The alkylamine concentration also affects the overall CdTe formation kinetics, as evidenced by the temporal evolution of the absorption spectra for different alkylamine concentrations (Figures S6 and S7). The NCs obtained at [DDA] < 10 mM are in the zero-dimensional quantum confinement regime, and therefore their size can be estimated from their lowest energy absorption peak using sizing curves for CdTe QDs.^30^ This analysis indicates that CdTe QDs of 2 nm diameter take 48 h to grow at [DDA] = 3.4 mM and only 90 s at [DDA] = 17 mM (Figure S7), clearly illustrating the impact of the [DDA] on the CdTe formation rates. Absorption spectroscopy has been often used to quantitatively follow the formation of colloidal QDs and nanosheets,^39,40^ yielding information over the temporal evolution of both the size and the concentration of the NCs, provided the size dependences of the peak position and molar absorption coefficient of the lowest energy exciton transition are known. In the present case, however, this approach is inadequate to quantitatively analyze the formation rates of the CdTe NCs for two reasons. First, as the reaction progresses CdTe nanostructures with different sizes and shapes are sequentially formed, which complicates the analysis since the position and oscillator strength of the lowest energy absorption peak are strongly dependent on both the size and shape of semiconductor NCs. Second, the molar absorption coefficients of the transitions involved are unknown. The absorbance at 300 nm (4.13 eV) is better suited to follow the CdTe formation rates since the density of states at such high energies above the band gap is expected to be (closer to) bulk-like^30^ and therefore is less affected by size- and shape-dependent quantum confinement effects. As a result, the absorbance at 300 nm should be proportional to the volume fraction of solid CdTe in solution, regardless of the size, shape, and concentration of the NCs. One should however bear in mind that this expectation is not strictly valid for the ultrathin CdTe MSNWs investigated here since they experience an extremely strong 1D-quantum confinement (their diameter ranges from 5% to 7.5% of the exciton Bohr diameter of CdTe). Nevertheless, the absorbance at 300 nm is a more reliable metric to follow the CdTe formation rates for different [DDA] than the absorbance of the lowest energy absorption transition, even if the observed trends are not strictly quantitative. A more thorough analysis of the temporal evolution of the absorption spectra is carried out in sections 3.4 and 3.5, aiming at unravelling the dynamics associated with each different MSNW species. In the present section, we will focus on the overall CdTe formation rates, regardless of the size and shape of the product NCs.

The temporal evolution of the absorbance at 300 nm of the CdTe products obtained in the low (0–84 mM) and high (0.29–2.04 M) DDA concentration ranges are presented in Figures 2b and S9, respectively. Figure S10 shows the trends for selected time points (30 s, 2 min, 14 min, 24 h, 48 h) over the entire concentration range investigated (1.7 mM to 2.04 M). For comparison, the data for 0.29 M OTA are included in all three figures. The trends summarized in Figure 2b clearly demonstrate that primary alkylamines significantly speed up the CdTe formation rates at room temperature. However, they are not essential components of the reaction, given that CdTe also forms in their absence, although at a much slower rate (Figure 2b). Tertiary alkylamines, such as OTA, also speed up the CdTe formation rates, although to a lesser extent than primary alkylamines (Figure 2b, S9, S10). It is interesting to note that the impact of primary alkylamines (RNH_2_) on the CdTe formation rate is strongly concentration- and time-dependent (Figure 2b, S9, and S10), being roughly first-order in the low concentration regime (<10 mM) at short reaction times (≤12 min) and decreasing rapidly as [RNH_2_] and the reaction time increase. Intriguingly, the trends change dramatically above the critical [RNH_2_] (0.1 M) when the different CdTe MSNW species become the sole reaction products, with the absorbance at 300 nm decreasing with [RNH_2_] at early reaction times and increasing with [RNH_2_] at later times. The trend at later times can be rationalized by considering that the contribution of the NW-450 MSNWs to the absorption spectra decreases with increasing [RNH_2_]. A closer inspection of the absorption spectra of NW-450 (see, e.g., 84 mM in Figure 2a, or Figure S6a) shows that this species has a different spectral shape below 350 nm, which may originate from shifting of the oscillator strength from higher energy to lower energy transitions, thereby decreasing the absorbance at 300 nm, in comparison with the other two MSNW species. This implies that for a quantitative analysis of the evolution of the relative population of each MSNW species, one must consider the entire absorption spectrum, rather than a single wavelength. Such an analysis will be carried out in section 3.5. The trend at early times also has important implications, which we will discuss below.

The formation of colloidal NCs of semiconductors consists of a chain of consecutive elementary kinetic steps: (i) the prenucleation period, in which monomer formation and assembly of subcritical nuclei occur, (ii) nucleation and (iii) growth.^3^ In the case of the CdTe MSNWs investigated in the present work, growth occurs in a stepwise fashion, and therefore the growth stage can better be described as two sequential and intertwined steps: (iii) growth in length of one of the MSNW species and (iv) interconversion between different MSNW species. Considering that the spectral signatures of CdTe monomer units are above 4.1 eV, the monomer conversion rates will contribute to the observed trends only if they are rate-limiting. This is clearly not the case above the critical [RNH_2_] since the volume fraction of solid CdTe at early reaction times in this concentration range decreases with increasing [RNH_2_], in contradiction with the fact that alkylamines are involved in the reaction mechanism leading to the formation of CdTe from Cd-oleate and TOP-Te, as demonstrated by the trends in the low [RNH_2_] range and discussed in detail in section 3.3 below. We can thus conclude that the observed trends imply that either nucleation or growth are rate-limiting and become slower with increasing [RNH_2_]. To identify which of these two elementary kinetic steps is rate-limiting, additional experiments are needed, which will be discussed in section 3.4 below. Moreover, the observations discussed above (Figures 2 and S4–S10) imply that primary alkylamines are essential in the room temperature formation of ultrathin CdTe MSNWs and have multiple roles at different stages of the reaction, depending on their concentration. Nevertheless, alkylamines interact with other components of the reaction system, and therefore their roles cannot be understood in isolation, as will be clear in the following section.

### 3.3 Role of Alkylphosphines in the Formation of CdTe Magic-Size Nanowires

Alkylphosphines enter the reaction system as part of the tellurium precursor, which is obtained by dissolving elemental tellurium in trioctylphosphine (TOP). The resulting TOP-Te complex is the limiting reagent (Cd/Te = 2) and is used in the reaction at a concentration of 1.67 mM, which results in a TOP concentration of 75 mM. Nevertheless, in the absence of diphenylphosphine (DPP), the reaction kinetics are very slow and strongly dependent on the batch of TOP used, with some batches resulting in no CdTe formation at all at room temperature. This is likely because commercial TOP commonly contains significant concentrations of secondary alkylphosphines (e.g., dioctylphosphine),^3,41,42^ which have been shown to be crucial in the formation of NCs of II–VI and IV–VI semiconductors using solutions of the chalcogen in TOP as precursors.^43,44^

**Figure 3:**
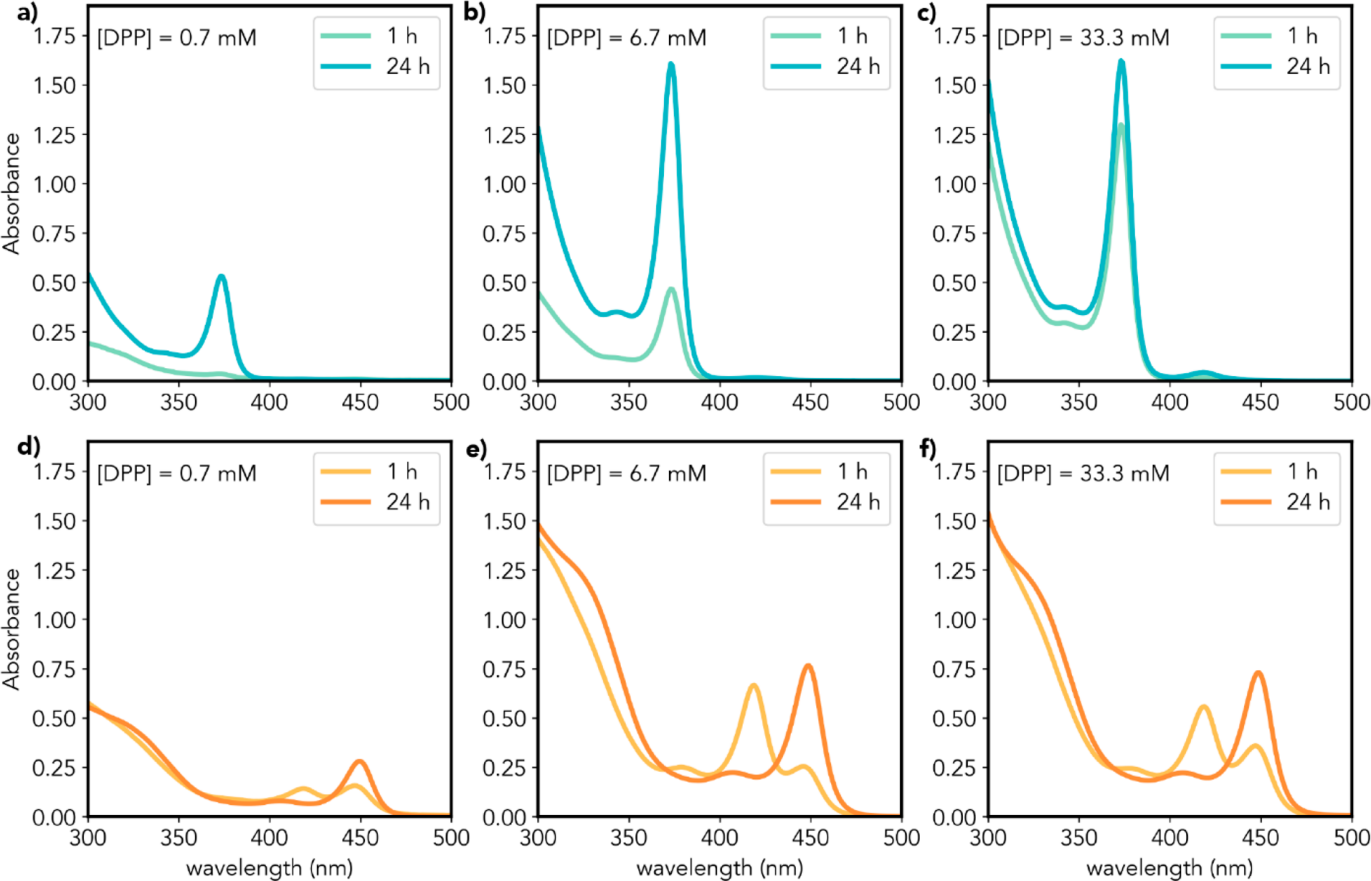
Absorption spectra of the CdTe products obtained after 1 and 24 h of reaction for (a–c) 2.04 M DDA and (d–f) 0.29 M DDA with three different concentrations of diphenylphosphine (DPP): 0.7 mM (a, d), 6.7 mM (b, e), and 33.3 mM (c, f). All other reaction parameters and dilution factors are the same.

In the present case, it is evident that DPP has an essential role in the reaction leading to the formation of CdTe MSNWs from Cd(oleate)_2_ and TOP-Te, since the reaction rates are faster with increasing DPP concentration (Figures 3 and S11). Further, for [DPP] leading to DPP/Te ≥ 2, the same absorption spectra and final absorbance are observed after sufficiently long reaction times (≥24 h) regardless of [DPP], implying that the concentration and nature of the CdTe MSNWs formed are the same. Interestingly, for DPP/Te smaller than 2 the final absorbance depends linearly on the DPP concentration, implying that DPP is quantitatively consumed in the reaction. These observations imply that DPP affects only the CdTe monomer formation rates, while the nature of the CdTe MSNW species formed are dictated solely by the concentration of the primary alkylamine (Figures 3 and S11). It is worth noting that the DPP impact on the reaction kinetics is modulated by the primary alkylamine concentration, being more pronounced at lower amine concentrations. This is clearly demonstrated by Figure 3, which shows that the CdTe volume fractions after 1 h of reaction for 2.04 M DDA are 5%, 29% and 83% of the final (i.e., after 24 h) under, respectively, 0.7, 6.7, and 33.3 mM DPP. In contrast, for 0.29 M DDA, the CdTe volume fraction after 1 h is already 92% of the final, even for DPP/Te smaller than 2. For higher [DPP] at lower [DDA], the reaction after 1 h consists primarily of the interconversion of NW-418 MSNWs into NW-450 MSNWs. This is consistent with the observations discussed in section 3.2 above and shows that DPP affects primarily the CdTe monomer formation rates, while the primary alkylamine affects also the nucleation, growth, and interconversion rates. It is also clear that the DPP impact is modulated by interaction with primary alkylamines.

The synthesis of colloidal metal telluride NCs is less well-developed than that of the selenide and sulfide counterparts. As a result, the reaction mechanisms leading to their formation have been scarcely studied.^43,44^ It is likely that the mechanism involved in the room temperature formation of CdTe MSNWs from Cd(oleate)_2_ and TOP-Te in the presence of DPP and primary alkylamines in toluene is analogous to that previously reported by Cossairt and Owen for the formation of CdSe magic-size clusters from Cd-benzoate and DPP-selenide in the presence of dodecylamine at 45 °C in toluene (Figure S12).^14^ The first step of this mechanism (viz., 2 DPP-Se + DDA → [DPPSe_2_]^−^ + DDA^+^ + DPP, Figure S12) is fast and quantitative also at room temperature^14^ and would thus be operative under the conditions prevalent in our work. This mechanism explains well our observations, including the fact that a DPP/Te ≥ 2 is needed. It is known that an equilibrium between the free tertiary alkylphosphine and Te^0^ is established in solutions of tertiary phosphine tellurides.^43^ It is thus plausible that tellurium transfer from TOP-Te to DPP forming DPP-Te would occur in situ. Given that according to the mechanism proposed in ref (14) (Figure S12) DPP-Te should quickly react with the primary alkylamine, the Te transfer equilibrium should be shifted toward DPP-Te and should be dependent on the DPP concentration for DPP/Te < 2.

We note that dialkylphosphines are more powerful reductants than their tertiary analogues. To understand whether this characteristic is critical for the reaction mechanism, DPP was replaced by a strong reducing agent of a different chemical nature, LiEt_3_BH (Super-Hydride). It has been shown that the addition of Super-Hydride to tertiary alkylphosphine tellurides in apolar solvents produces oligotelluride anions (Te_*n*_^2–^, *n* = 1, 2, 3), the speciation of which depends on the amount of hydride reducing agent (*n* = 1, 2, 3 for, respectively 2, 1, and 0.7 equiv of Super-Hydride).^45^ Oligotellurides have been shown to slow down the formation of ZnTe NCs and affect their shapes.^45^ Interestingly, in the present case, the reaction evolved in a similar way and showed the same dependence with respect to the DDA concentration (Figure S13). This implies that the reducing power of secondary phosphines is indeed a critical parameter in the room temperature formation of CdTe monomers from Cd(oleate)_2_ and TOP-Te, and suggests that negatively charged Te species are crucial in the room temperature formation of CdTe magic-size nanowires. Importantly, these observations support the conclusion that the diameter of the CdTe MSNWs is dictated entirely by the concentration of the primary alkylamines. The significance of these observations will be discussed in more detail below.

### 3.4 Formation Mechanism of CdTe Magic-Size Nanowires

**Figure 4:**
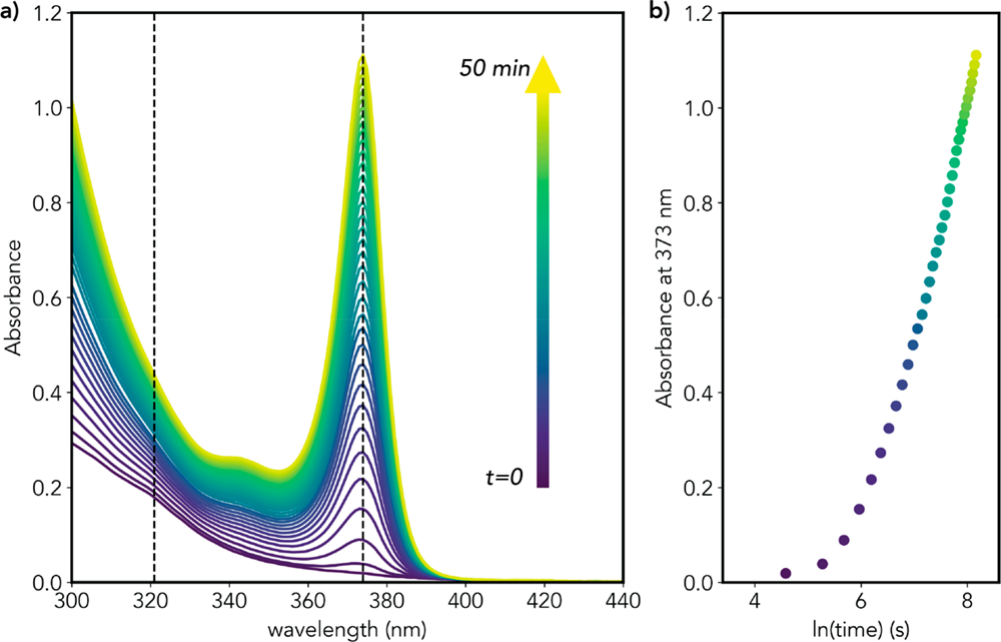
(a) In-situ temporal evolution of the absorption spectra of a CdTe reaction mixture containing 2.04 M DDA during the first 54 min of reaction. The spectra were acquired every 1.5 min in cuvettes with a 1 mm optical path using the neat reaction mixture. The DPP concentration was 3.34 mM ([DPP]/[Te] = 2). The dashed lines mark the positions of two relevant spectral features at 320 and 373 nm. (b) Temporal evolution of the absorbance at 373 nm, which is characteristic of the thinnest magic-size nanowire NW-373.

As shown above, three different species of CdTe MSNWs can be obtained from Cd(oleate)_2_ and TOP-Te at room temperature, provided sufficiently high concentrations of a primary alkylamine (RNH_2_) and DPP are present (Figures 1, 3, and S6). The population of each different CdTe MSNW species is determined entirely by the primary alkylamine concentration [RNH_2_] so that single-species are only obtained at specific concentrations, while mixtures of two different MSNW species are obtained at concentrations intermediate between the specific ones (Figures 1, 3, and S6). At [RNH_2_]< 2 M, formation of MSNWs is accompanied by quantized growth (Figure S6), which is equivalent to interconversion between MSNW species and may be accompanied or followed by growth in length. The formation of ultrathin CdTe MSNWs thus consists of a chain of consecutive elementary kinetic steps: (i) monomer formation, (ii) nucleation, (iii) growth in length, and (iv) interconversion. As discussed above, the latter two steps are strongly intertwined. In this section, we will discuss each of the elementary kinetic steps, except for the latter, which will be addressed in section 3.5 below. To avoid the interference of concomitant interconversion between different species, we followed the temporal evolution of the absorption spectra of a CdTe reaction mixture containing 2.04 M DDA, in which only the thinnest MSNWs (NW-373) are stable (Figure 4). To facilitate the observation of the early stages of the reaction, the kinetics were slowed down by using the minimum amount of DPP required (i.e., DPP/Te = 2). We note that NW-373 MSNWs are the first to form, regardless of [RNH_2_]. The formation mechanism discussed below is thus valid for all three species of CdTe MSNWs since the thicker ones (NW-418 and NW-450) sequentially evolve from NW-373.

Figure 4 shows that at very short reaction times (≤30 s), the characteristic absorption peak of NW-373 (373 nm) is not yet observable, while a weak and broad peak at 320 nm is present. This peak quickly fades over time and is no longer observable after ∼10 min of reaction. The 373 nm peak appears already after ∼1 min, coexisting with the 320 nm peak. After ∼10 min of reaction, the characteristic absorption transitions of NW-373 MSNWs become the only observable spectral features and increase steadily over time. The peak at 320 nm must be related to either monomer formation or nucleation since it precedes the appearance of the spectral features associated with NW-373. Considering that this peak is no longer observable after ∼10 min, while growth proceeds for hours without any spectral change other than the increase of the absorbance of the transitions associated with NW-373, we assign it to CdTe nuclei. If the 320 nm peak was due to CdTe monomers, its intensity should still change during the growth period, in contrast with the experimental observations. The fact that the 320 nm and the 373 nm peaks coexist at the early stages of the reaction (≤10 min) implies that the rate-limiting step is the growth of the MSNWs in length because otherwise the absorption transitions associated with the nuclei would not be observable since they would be quickly consumed in the growth step. This assignment is consistent with the observations discussed in section 3.2 above. As a result, the nucleation stage extends over a relatively wide time window, starting when the critical monomer supersaturation is reached and continuing while CdTe monomers are replenished at a rate that exceeds the growth rate. As the concentration of CdTe nuclei increases, the monomer consumption rate due to growth accelerates, eventually bringing the CdTe monomer concentration to levels that no longer sustain nucleation. The growth then proceeds in the absence of nucleation, and thereby the absorption peak associated with the CdTe nuclei eventually disappears. Extended nucleation has been recently observed for colloidal CdSe QDs synthesized from Cd(oleate)_2_ and TOP-Se and shown to promote size focusing due to reaction-limited size-dependent growth rates.^39^ As will be discussed below, the growth of the CdTe MSNWs is likely also reaction-limited.

The precursor to monomer conversion has often been observed to be rate-limiting in the formation of colloidal semiconductor NCs.^3,46−49^ Nevertheless, this is not the case in the present work, even though the reaction is carried out at room temperature. As discussed above (sections 3.2 and 3.3), the combination of a primary alkylamine and DPP significantly enhances the room temperature formation rates of CdTe from Cd(oleate)_2_ and TOP-Te. It is worth noting that secondary and tertiary alkylamines also accelerate the CdTe formation rate, albeit to a much lesser extent than their primary counterparts, allowing the critical supersaturation limit required for CdTe nucleation to be exceeded after sufficiently long times (section 3.2). However, similarly to when only DPP is used (i.e., amines are absent), secondary and tertiary alkylamines yield only CdTe QDs, regardless of the temperature. This is in striking contrast with primary alkylamines, which at room temperature yield only ultrathin magic-size nanowires at any [RNH_2_] above 0.1 M, and ultrathin twisted NCs at [RNH_2_] as low as 3.4 mM (Figure 2).

At sufficiently high [RNH_2_] (≥0.1 M), CdTe QDs are only obtained at reaction temperatures above 100 °C, while temperatures at 40–100 °C favor the formation of nanoribbons or nanosheets, depending on the temperature and reaction time (Figure S14). In this context, it is interesting to note that Talapin and co-workers recently reported that ZnSe MSNSs of progressively higher dimensionality (0D, 1D, and 2D) are sequentially formed in a heat-up synthesis protocol depending on the final reaction temperature, with higher temperatures favoring higher dimensionalities.^20^ The study of the influence of the reaction temperature lies beyond the scope of this paper and will be the subject of follow-up work. Nevertheless, these observations imply that in the presence of primary alkylamines CdTe nucleation and growth follow fundamentally different paths with respect to those available in their absence, suggesting that primary alkylamines impose prohibitively high energy barriers to nucleation and growth of 0D CdTe NCs while favoring the formation of anisotropic 1D NCs. As will be discussed below, we attribute this to the combined effects of a reaction mechanism that leads to fast monomer formation and the ability of primary alkylamines to form dense, ordered monolayers stabilized by van der Waals interactions.

As demonstrated in previous works, the formation of magic-size clusters (MSCs) is favored by synthesis protocols in which the monomer formation is fast and the constituent elements of the MSCs are already in their final oxidation state in the precursors.^13,14,50,51^ Moreover, reaction protocols involving negatively charged Te_*n*_^2–^ species have been shown to yield not only metal telluride MSCs but also magic-size nanosheets at sufficiently high temperatures.^42,52−54^ We thus propose that the reaction between Cd(oleate)_2_ and TOP-Te at room temperature in the presence of primary alkylamines and DPP leads to the fast formation of CdTe monomers. Considering that monomers are defined as the smallest units capable of inducing crystal nucleation,^3^ we propose that the CdTe monomers consist of [(RNH_2_)_*n*_(CdTe)] complexes. This assignment is consistent with the reaction mechanism discussed above (section 3.3, Figure S12)^14,43^ but should be verified by further studies, which are nevertheless beyond the scope of this work. The fast formation of (RNH_2_)_*n*_(CdTe) monomer units would quickly yield very high oversaturations, thereby favoring the formation of MSCs.^13,14,50,51^ MSCs follow a quantized growth pathway through which increasingly larger [ME]_*n*_ (*n* = 2, 4, 13, 19, 33, 66, 84) clusters are sequentially formed.^13^ This process can be rationalized in terms of nonclassical nucleation models, in which each subsequent [ME]_*n*_ MSC occupies a local free-energy minimum in the progression from monomers to crystals.^13^ The depths of these free energy minima are dictated by the reaction conditions (i.e., temperature, monomer supersaturation, nature, and concentration of adjuvant species, such as ligands).^13^ Therefore, different conditions will stabilize different MSC sizes, halting the quantized growth process when the most stable [ME]_*n*_ MSC is reached.^13^ They can thus accumulate in the reaction medium, acting as kinetically persistent intermediates for subsequent processes that have high activation energies but lead to high free-energy gain.

We suggest that under the conditions prevalent in our experiments (viz., fast CdTe monomer formation, high oversaturation, high [RNH_2_], low temperature), CdTe MSCs are formed that act as nonclassical nuclei for the growth of ultrathin CdTe MSNWs. The fact that NW-373 MSNWs are the first to form regardless of [RNH_2_] implies that they have the lowest energy barrier for nucleation. The diameter of these CdTe MSCs is estimated as 1.3 nm, using the spectral position of the absorption peak attributed to CdTe nuclei (viz., 320 nm, Figure 4) and a previously published sizing curve for CdTe QDs.^30^ This size corresponds to a CdTe QD with 15 CdTe units (unit cell volume = 1.568 × 10^–22^ cm^3^, *z* = 2 for wurtzite CdTe),^30^ which is in excellent agreement with one of the MSC compositions (*n* = 13), especially considering the approximations involved in the estimate (spherical QD with bulk density). We thus conclude that [CdTe]_13_ MSCs are formed at early reaction stages and act as nuclei for the growth of NW-373 MSNWs. It is interesting to note that recent experimental and theoretical work has shown that in the presence of primary alkylamines [CdSe]_13_ MSCs adopt a tubular conformation that maximizes the number of Cd directly bound to NH_2_ groups.^55^ It is also noteworthy that theoretical modeling of the [CdSe]_13_ tubular structures suggests that they are directional, with one Se atom and one Cd atom positioned at each end of the long axis, thereby being highly polar (dipole moment of ∼27 D in toluene).^55^ We argue that such a tubular configuration would make (RNH_2_)_*n*_([CdTe]_13_) MSCs very favorable as nuclei for nanowires. We note that MSCs have been shown to function as monomer reservoirs under suitable conditions, redissolving to feed the growth of nanocrystals.^56^ This scenario, however, can be ruled out in the present case because growth of the MSNWs proceeds long after the spectral signatures attributed to the MSCs have disappeared. The same argument can be used to exclude the possibility that NW-373 MSNWs form by oriented attachment of CdTe MSCs. Oriented attachment has been often invoked to explain the formation of nanowires of II–VI semiconductors^13,21,35−37^ and is evidenced by the fact that nanowire growth no longer proceeds after the preexisting building blocks (MSCs or nanocrystals) have been depleted, in striking contrast with our observations.

Primary alkylamines have been observed to form stable self-assembled monolayers on the surfaces of a variety of materials, both in the bulk (e.g., mica,^57^ gold,^58^ copper^59^) and nanocrystalline forms.^3,22,58,59^ In the case of solution-grown nanocrystals, the packing of RNH_2_ molecules at the nanocrystal facets has been demonstrated to have a critical shape-directing effect.^3,22^ We propose that primary alkylamines (RNH_2_) promote 1D growth by forming densely packed monolayers at the surface of the growing CdTe MSNWs. The dense RNH_2_ monolayers exert their 1D-directive effect both by blocking access to the lateral surfaces of the nanowires, thereby preventing isotropic growth, and by providing additional stability to the MSNWs due to the van der Waals interactions between the alkyl chains. This explains why CdTe MSNWs only form above a critical [RNH_2_]. The fact that the growth is the rate-limiting step can be rationalized by considering that the CdTe monomers are bound to RNH_2_ molecules which must leave prior to incorporation of the monomer unit into the growing nanowire, making the process reaction-limited. Further, RNH_2_ molecules are also transiently bound to the growing (002) facets of the nanowire and must be removed to allow access to the surface sites. This explains why the growth rates decrease with increasing [RNH_2_] (see section 3.2 above and section 3.5 below) since a higher [RNH_2_] decreases the accessibility of the growing facets of the CdTe nanowire. We note that primary alkylamines have been proposed to promote the formation of MSCs, nanoribbons, and nanosheets through the formation of mesophases that act as soft templates.^21,60,61^ However, SAXS experiments have not provided any evidence for the presence of mesophases in the reaction mixtures investigated in our work. The 1D-directive effect of primary alkylamines observed in the present work is thus attributed to a synergistic and dynamic interaction between the growing CdTe MSNWs and RNH_2_ molecules bound to both the surface of the MSNWs and to the CdTe monomers so that the formation of the RNH_2_ monolayer at the surface of the growing CdTe MSNWs accounts for a significant fraction of the free-energy gain during the growth, thereby providing a large contribution to the driving force. The ultrathin CdTe MSNWs can thus be seen as hybrid organic–inorganic nanostructures since their free-energy is determined by both the CdTe nanowires and the RNH_2_ monolayers coating their surface. The significance of this notion will be discussed in more detail in the following two sections.

### 3.5 Dynamics of Formation and Interconversion of CdTe Magic-Size Nanowires

In the sections above, the first three kinetic steps of the formation of ultrathin CdTe MSNWs (monomer formation, nucleation, and growth in length) were discussed in detail for the specific case of NW-373 MSNWs. The formation mechanism discussed in section 3.4 is however valid for all three CdTe MSNW species, since the thicker ones (NW-418 and NW-450) evolve from NW-373, which are the first to form at any [RNH_2_] above the critical one. This is clearly illustrated by Figures S6 and 3, which also demonstrate that growth in length and interconversion are strongly intertwined since the evolution of the reaction is characterized both by an increase of the absorbance of specific transitions due to an increase in length and/or concentration of specific MSNW species and by discrete spectral jumps due to quantized growth of the MSNWs (interconversion from thinner to thicker MSNW species). To gain insight into the growth and interconversion dynamics of the different CdTe MSNW species, the entire absorption spectra must be quantitatively analyzed. The temporal evolution of the absorption spectra at single wavelengths is insufficient for this purpose since the characteristic absorption transitions of thinner MSNW species partially overlap with higher energy transitions of thicker ones, while the absorbance at 300 nm is proportional to the CdTe volume fraction regardless of the size and shape of the absorbing nanostructures.

To develop a procedure to quantitatively analyze the contribution of each CdTe MSNW species to the absorption spectra of the reaction products, we explored the fact that the MSNWs remain responsive to the RNH_2_ concentration, even after the reaction has reached completion at a certain [RNH_2_] (see Supporting Information, section S3 for details). For example, a colloidal suspension initially containing single-species CdTe NW-373 MSNWs at 2.04 M DDA will evolve to single-species CdTe NW-450 MSNWs if the DDA concentration is decreased to 0.29 M (Figure S15a). Most interestingly, the conversion of NW-373 into NW-450 is quantitative, without the formation of any other byproduct, and the initial and final concentrations of CdTe MSNWs are the same as those obtained by direct synthesis of the two different species (Figure S15a,b). We can thus conclude that the amount of CdTe formed at reaction times ≥ 24 h is the same, regardless of [RNH_2_]. These observations imply that the absorption spectra of single-species MSNWs correspond to the same amounts of CdTe (Figure S15c) and that the total amount of CdTe distributed over all MSNWs formed in the reaction mixture after 24 h is the same for all [DDA] above 0.1 M, regardless of the dimensions of the nanowires. Given that only three different species of MSNWs can be formed and that each of them is characterized by a unique absorption spectrum, this implies that linear combinations of the spectra of single-species MSNWs (labeled P1, P2, P3 in Figure S15c) can be used to reproduce the absorption spectra of any suspension containing the three MSNW species in any ratio (Figure S15d). Most importantly, the coefficients of these linear combinations are proportional to the partition of the total amount of CdTe available in the reaction medium between the different species of MSNWs. Considering that the characteristic absorption spectrum of a MSNW is dictated only by its diameter (the confinement dimension), while the total absorbance is determined by the total volume of absorbing material, it follows that the coefficients obtained by fitting the absorption spectra as linear combinations of the spectra of single-species MSNWs are equivalent to the relative volume fraction of each MSNW species. The fitting coefficients thus give the relative amount of CdTe locked into each of the different MSNW species but provide no information concerning the concentration (i.e., number density) of each species, given that the total absorption cross-section of one longer MSNW with length *nL* is in principle identical to that of *n* shorter MSNWs with length *L*.

**Figure 5:**
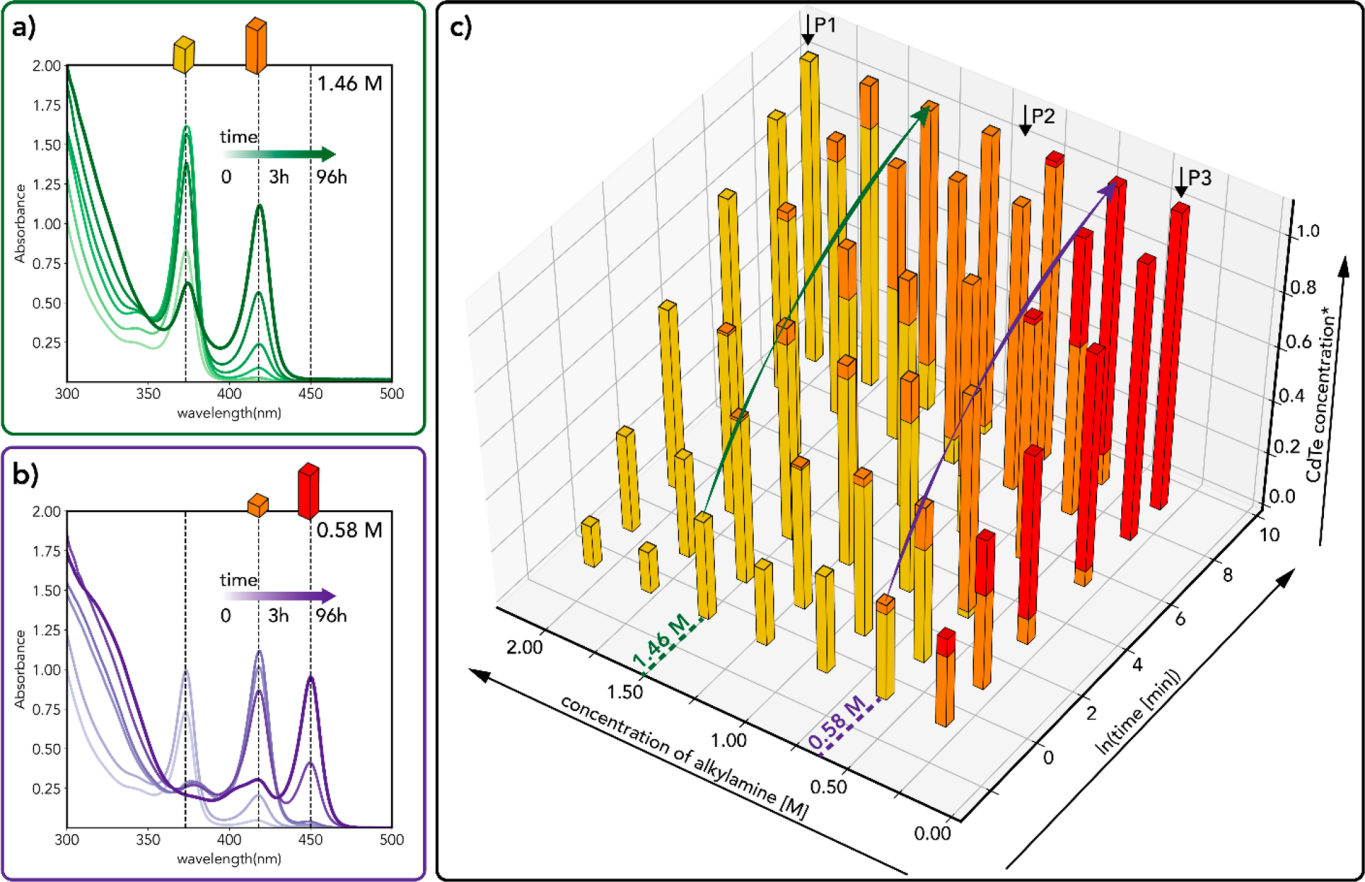
(a, b) Temporal evolution of the absorption spectra of the products obtained during 96 h of reaction for two different DDA concentrations: 1.46 M (a) and 0.58 M (b). The first measurement (indicated by “0” in the legends) was carried out ∼30 s after the preparation of the reaction mixture. The CdTe volume fractions of each magic-size nanowire (MSNW) species after 96 h (4 days) are schematically represented by colored blocks. Yellow represents the thinnest MSNWs (NW-373), orange represents the intermediate MSNWs (NW-418), and red represents the thickest MSNWs (NW-450). The height of the blocks is proportional to the CdTe volume fraction of each MSNW species, which were obtained following the procedure described in the Supporting Information (section S3). The total volume fraction of CdTe (“CdTe concentration”) after 4 days is normalized to 1. (c) 3D histogram of the temporal evolution of the CdTe volume fraction of each MSNW species for different DDA concentrations, using the same color code and procedure as in in panels (a, b). The absorption spectra and fit results used to construct the histogram are provided in the Supporting Information (Figures S16–S23). The green and blue arrows indicate the evolution for 0.58 and 1.46 M DDA. The black arrows mark the DDA concentrations that yield single species MSNWs (2.04, 0.87, and 0.29 mM, for NW-373, NW-418, and NW-450, respectively) and are labeled in the same way as their respective absorption spectra in Figures S15–S23.

**Figure 6:**
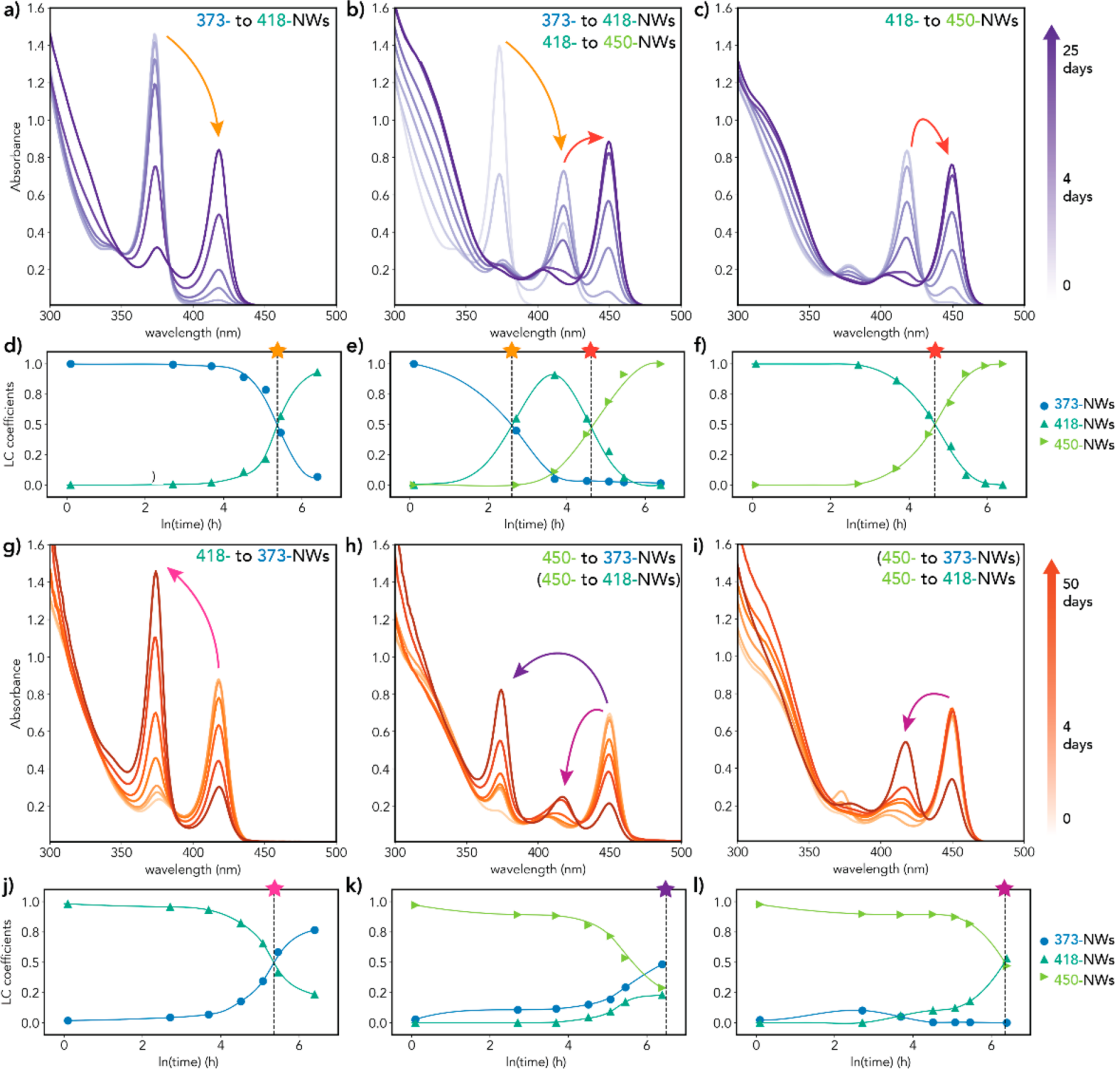
Temporal evolution of the absorption spectra of solutions initially containing single-species CdTe MSNWs at their original DDA concentration ([DDA]_0_) after their dilution to a DDA concentration ([DDA]_new_) that stabilizes a different MSNW species. The absorption spectra shown in panels (a–c) and (g–h) were fitted to linear combinations of the spectra of single-species MSNWs following the procedure described in section S3. The temporal evolution of the linear combination (LC) coefficients for each sample is shown in panels (d–f) and (j–l). The arrows in panels (a–c) and (g–i) indicate the direction of the interconversion process given at the top of the panel. Processes between brackets occur in parallel with the main interconversion process, which is given without brackets. The color code is the same in all panels. The stars and dashed lines in panels (d–f) and (j–l) mark the reaction half-life *t*_1/2_ (i.e., time at which the concentration of the original species has reached 50% of its initial value). The *t*_1/2_ values are collected in Table 1. (a, d) Original MSNWs: NW-373, [DDA]_0_ = 2.04 M, [DDA]_new_ = 0.87 M; (b, e) Original MSNWs: NW-373, [DDA]_0_ = 2.04 M, [DDA]_new_ = 0.29 M; (c, f) original MSNWs: NW-418, [DDA]_0_ = 0.87 M, [DDA]_new_ = 0.29 M; (g, j) original MSNWs: NW-418, [DDA]_0_ = 0.87 M, [DDA]_new_ = 2.04 M; (h, k) original MSNWs: NW-450, [DDA]_0_ = 0.29 M, [DDA]_new_ = 2.04 M; (i, l) original MSNWs: NW-450, [DDA]_0_ = 0.29 M, [DDA]_new_ = 0.87 M.

This procedure allowed us to quantitatively analyze the growth and interconversion dynamics of the different CdTe MSNW species both during reactions carried out at different [DDA] (Figure 5) and after postsynthetic changes in [DDA] (Figure 6). We will first discuss the MSNW formation dynamics during the reaction and subsequently address their transformation and interconversion in response to postsynthetic changes in the [RNH_2_]. A mechanism explaining the formation and interconversion of the CdTe MSNWs both during and after the reaction will be discussed in section 3.6 below.

Figure 5 shows that NW-373 are the first to form regardless of [DDA] but convert to NW-418 at [DDA] < 2 M. Moreover, the NW-373 to NW-418 interconversion rates accelerate with decreasing [DDA] (Figures 5c, S6, S16–S23). For example, it takes 24 h for the fraction of NW-418 to reach 7% at 1.75 M DDA (Figures 5c and S22) and less than 30 s at 0.58 M DDA (NW-418 fraction is 9% at 30 s, Figures 5c and S17). At 0.87 M, DDA NW-418 MSNWs become the only species present after ∼24 h. At [DDA] < 0.87 M, NW-418 become unstable, converting into NW-450 (Figure 5c, S6, S16–S18) at rates that are inversely proportional to [DDA] so that at 0.29 M DDA NW-450 become the only MSNW species present after ∼24 h. The acceleration of the NW-418 to NW-450 interconversion with decreasing [DDA] is clearly illustrated in Figure S24, which shows the in situ temporal evolution of the absorption spectra of reaction mixtures containing two different [DDA] that stabilize NW-450 as the final MSNW species (viz., 0.15 and 0.29 M, which result in isosbestic points after 6 and 47.5 min of reaction, respectively). Interestingly, the sum of the absorbances at 417 and 450 nm and the absorbance at 300 nm follow a similar trend in both cases, remaining constant throughout the reaction after an initial period of growth. Moreover, the final absorbance at 300 nm is the same for both [DDA]. These observations corroborate the conclusions presented above that the total amount of CdTe formed at sufficiently long reaction times is the same regardless of [RNH_2_] and is not affected by the interconversion between different MSNW species. They also demonstrate that the MSNW species interconvert directly into each other, without any observable intermediates or dissolution.

We turn now to the interconversion between different CdTe MSNW species in response to postsynthetic changes in [RNH_2_]. To investigate this intriguing process, we followed the temporal evolution of the absorption spectra of a series of CdTe MSNW samples subjected to [DDA] different from those at which they had originally been prepared (Figure 6). To this end, aliquots of neat reaction mixtures containing only one of the three CdTe MSNW species (NW-373, NW-418, or NW-450) at their original DDA concentration ([DDA]_0_ = 2.04, 0.87, or 0.29 M, respectively) were diluted in solutions of DDA in toluene, such that the resulting [DDA] ([DDA]_new_) was equal to one of the specific [DDA] that stabilizes a different MSNW species. It should be noted that the mixtures containing the original single-species CdTe MSNWs were allowed to react for 4 days prior to dilution to [DDA]_new_ to ensure that the MSNWs had reached their most stable configuration at [DDA]_0_. Moreover, the concentration of CdTe after dilution is the same in all cases, regardless of the original MSNW species and [DDA]_new_. The measurements were carried out in sealed 10 mm quartz cuvettes stored in a glovebox under N_2_ during the intervals between measurements. The absorption spectra were fitted to linear combinations of the spectra of single-species MSNWs following the procedure described in section S3 and used to construct Figure 5 above. An example of this analysis for the diluted MSNW samples is provided in Figure S25.

For example, a solution initially containing single-species CdTe NW-373 MSNWs ([DDA]_0_ = 2.04 M, see, e.g., Figure S23) was diluted with a solution of DDA in toluene so that the final DDA concentration ([DDA]_new_) was 0.29 M, which stabilizes single-species CdTe NW-450 MSNWs (e.g., Figure S16). In response to the change in [DDA], the NW-373 gradually transformed into NW-418 and subsequently into NW-450 so that after 25 days NW-450 was the only MSNW species present (Figures 6b,e and S25). It should be stressed that the postdilution transformations are a response to changes in the concentration of the primary alkylamine and not of the CdTe MSNWs. This is clearly demonstrated by the fact that the absorption spectrum remains unchanged if [DDA] after dilution of the neat reaction mixture is the same as the original one (i.e., ([DDA]_new_= ([DDA]_0_), despite the lower concentration of CdTe MSNWs. The temporal evolution of the linear combination coefficients shows that NW-373 were almost completely interconverted to NW-418 after ∼100 h with a reaction half-life *t*_1/2_ of 11 h, while the interconversion of the newly formed NW-418 into NW-450 occurred with a *t*_1/2_ of 110 h (Figures S25 and 6b,e, Table 1).

**Table 1:**
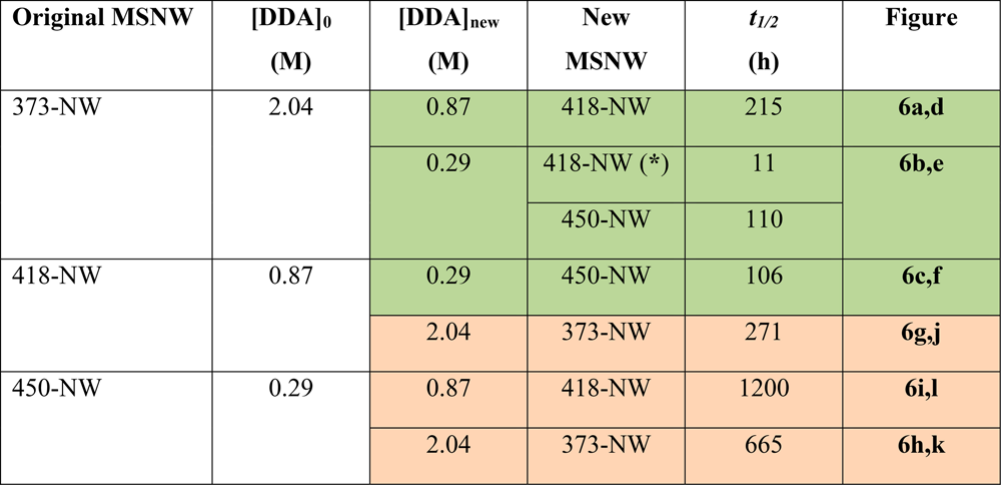
Summary of the Kinetics of the Interconversion between the Three Different CdTe MSNW Species Shown in Figure 6 of the Main Text^a^

a [DDA]_0_ = original DDA concentration prior to dilution of the neat reaction mixture containing one of the three CdTe MSNW species; [DDA]_new_ = DDA concentration after dilution; *t*_1/2_ = reaction half-life (i.e., time at which the concentration of the original MSNW species has reached 50% of its initial value). Eurymorphic (i.e., thinner to thicker) magic-diameter interconversions are highlighted in green, and leptomorphic (i.e., thicker to thinner) magic-diameter interconversions are highlighted in orange. *Intermediate on-route to NW-450.

Figure 6 shows that reduction of [DDA] induces sequential interconversion from thinner to thicker CdTe MSNWs (Figure 6a–f), while an increase in [DDA] leads to sequential interconversion from thicker to thinner CdTe MSNWs (Figure 6g–l). Given that the interconversion changes only the dimensions of the MSNWs, we will hereafter refer to these transformations as “eurymorphic” (from thinner to thicker) and “leptomorphic” (from thicker to thinner) magic-diameter transitions. Importantly, regardless of the direction of the interconversion (i.e., eury- or lepto-), the final stable MSNW species is entirely determined by the DDA concentration after dilution ([DDA]_new_). This is clearly demonstrated by an experiment in which equal volumes of solutions initially containing single-species CdTe NW-450 ([DDA]_0_ = 0.29 M) and CdTe NW-373 MSNWs ([DDA]_0_ = 2.04 M) were mixed yielding a solution with 1.16 M DDA (Figure S26). At this [DDA], NW-418 MSNWs are the most stable species (Figure S20). Consequently, NW-373 MSNWs undergo an eurymorphic interconversion to NW-418 MSNWs, while NW-450 MSNWs undergo a leptomorphic interconversion to NW-418 MSNWs. The rates for the eury- and leptomorphic interconversions are however very different: interconversion from NW-373 to NW-418 is complete after 1 day, while after 7 days the interconversion of NW-450 to NW-418 has just exceeded *t*_1/2_.

The much slower kinetics of the leptomorphic interconversions is also evident in the experiments summarized in Figure 6 and Table 1. For example, *t*_1/2_ is 271 h for the NW-418 to NW-373 leptomorphic interconversion (Figure 6g,j) and 215 h for the NW-373 to NW-418 eurymorphic interconversion (Figure 6a,d). The difference is even more dramatic when NW-450 is involved: *t*_1/2_ is 1200 h for the NW-450 to NW-418 interconversion (Figure 6i,l) and only 106 h for the NW-418 to NW-450 interconversion (Figure 6c,f). Moreover, the interconversion kinetics is faster for larger differences between [DDA]_new_ and [DDA]_0_, implying that the driving force for the interconversion is provided by Δ_[DDA]_. This is clearly illustrated by the NW-373 to NW-418 eurymorphic interconversion, which is much faster at [DDA]_new_ = 0.29 M (Figure 6b,e), where NW-418 forms as an intermediate *on route* to NW-450 (*t*_1/2_ = 11 h), than at [DDA]_new_ = 0.87 M (*t*_1/2_ = 215 h) (Figure 6a,d), despite the fact that NW-418 is the only MSNW species stable at 0.87 M. It is thus clear that the interconversion between the three CdTe MSNWs is entirely driven by [RNH_2_]. A mechanism to rationalize the dramatic impact of primary alkylamines on the stability of CdTe MSNWs will be discussed in section 3.6 below.

### 3.6 Mechanism for the Formation and Reversible Interconversion Between CdTe Magic-Size Nanowires

The results discussed above unambiguously demonstrate that primary alkylamines (RNH_2_) are essential in the formation of CdTe MSNWs from Cd(oleate)_2_ and TOP-Te at room temperature. Their crucial role in the monomer formation mechanism was discussed in section 3.3, while their decisive impact on the nucleation and growth of the MSNWs was discussed in section 3.4, where we proposed that the ultrathin CdTe MSNWs should be seen as hybrid organic–inorganic nanostructures. The 1D-directive effect of RNH_2_ was attributed to a synergistic and dynamic interaction between the growing CdTe MSNWs and RNH_2_ molecules bound to both the surface of the MSNWs and to the (CdTe) monomers so that the formation of the RNH_2_ monolayer provides a significant contribution to the free-energy gain during the growth. The fact that the thinnest MSNWs (NW-373, 0.7 ± 0.1 nm diameter) are always the first to form was rationalized in section 3.4, where we concluded that at sufficiently high [RNH_2_] (≥0.1 M) a new reaction pathway is created in which nucleation and continuous growth of QDs involve prohibitively high activation energies, while nucleation and growth of MSNWs becomes favorable. In section 3.5 above, it became evident that the NW-373 MSNWs are nevertheless unstable at [RNH_2_] below 2 M, interconverting to thicker MSNWs (NW-418, 0.9 ± 0.2 nm diameter). The NW-418 MSNWs also become unstable if [RNH_2_] is lower than 0.87 M, interconverting to NW-450 (1.1 ± 0.2 nm diameter). The MSNW species were observed to interconvert directly into each other, without any observable intermediates or dissolution. Interestingly, the thinner to thicker interconversion rates were observed to increase with decreasing [RNH_2_]. After interconversion at early stages of the reaction, thicker MSNWs grow further in length by incorporating CdTe monomers from solution. The final MSNW diameter is dictated entirely by [RNH_2_], with higher concentrations favoring thinner MSNWs. Intriguingly, CdTe MSNWs remain responsive to [RNH_2_], undergoing eurymorphic magic-diameter interconversions upon reduction of [RNH_2_] and leptomorphic magic-diameter interconversions upon increase of [RNH_2_] (Figure 6). Further, postsynthetic eurymorphic transitions were observed to be much faster than the leptomorphic analogues (Figure 6), but nevertheless slower than the eurymorphic interconversions observed during the formation of the MSNWs at [RNH_2_] < 2 M, when the initially formed NW-373 MSNWs transform into the species that are most stable at a given [RNH_2_] (Figure 5).

Transformation of MSNSs of II–VI semiconductors in response to changes in [RNH_2_] has been reported by several groups. Kasuya and co-workers observed that octylamine (OTA) capped CdSe MSNSs absorbing at 415 nm (assigned to [CdSe]_34_ MSCs) were converted to MSNSs absorbing at 350 nm by increasing [OTA] in a solution in toluene at room temperature.^15^ The transformation led to the formation of a precipitate, presumed to consist of MSCs with 1.2 nm diameter.^15^ Buhro and co-workers reported similar spectral transformations for RNH_2_ derivatives of stoichiometric ZnSe, CdSe, and CdTe MSNSs and interpreted them as conversion of [ME]_34_ MSCs to [ME]_13_ MSCs.^16^ The transformation process was significantly accelerated at high [RNH_2_].^16^ The results reported by Yu and co-workers are particularly relevant in the context of our work because the absorption spectra observed by these authors for three different species of CdTe MSNSs (labeled sMSC-371, sMSC-417, and sMSC-448 in the original work)^17,18^ are extremely similar to those observed in the present work for, respectively, NW-373, NW-418 and NW-450 MSNWs. Moreover, they also observed interconversion between the different CdTe MSNSs in response to changes in [RNH_2_], with higher concentrations favoring the species that absorb at higher energies.^17,18^ Yu and co-workers interpreted their observations under the assumption that the species responsible for the absorption spectra were CdTe MSCs and therefore attributed the transformations to isomerization between MSCs with the same number of atoms but different configurations.^17,18^ However, this interpretation can be ruled out in the present work because the species responsible for the absorption spectra are undoubtedly ultrathin CdTe magic-size nanowires with one out of three possible diameters (viz., 0.7 ± 0.1 nm, 0.9 ± 0.2 nm, or 1.1 ± 0.2 nm).

The interconversion between different magic-size nanostructures observed in the present work can be rationalized by considering the hybrid organic–inorganic nature of the CdTe MSNWs, which implies that their stability is synergistically determined both by the CdTe nanowire and by the monolayer of primary alkylamines that self-assembles on the surface of the nanowires during their growth. The formation of nanowires is kinetically driven because one-dimensional (1D) growth has much lower activation energies than two- and three-dimensional growth, especially if the nanowire diameter is smaller than the critical radius for island nucleation.^20,62^ Under reaction-limited conditions, the formation and growth of ultrathin CdTe 1D-nanowires thus offer the fastest pathway toward decreasing the total free-energy of the system, both by lowering the chemical potential of the CdTe monomers in solution and by forming Cd–Te solid-state bonds. Nevertheless, the nanowires will tend to evolve toward more isotropic shapes in order to minimize their surface free-energy through reduction of the surface to volume ratio.^3^ This tendency is however counteracted by the concomitant self-assembly of a monolayer of primary alkylamine molecules at the surface of the growing nanowire, which enhances the driving force for 1D-growth by conferring additional stability to the nanowires through both the formation of Cd-NH_2_ bonds, which lowers the surface free energy of the nanowires, and van der Waals interactions between neighboring alkyl chains, which are maximized in fully packed monolayers. The diameter of the product CdTe MSNWs is thus dictated by the balance between two opposing trends: maximization of the area of fully packed RNH_2_ monolayers, which favors the thinnest MSNWs (NW-373, 0.7 nm diameter), and minimization of the surface to volume ratio of the CdTe nanowires, which favors the thickest MSNWs (NW-450, 1.1 nm diameter). Given that the thermal energy available at room temperature is insufficient to allow the formation of nanowires thicker than 1.1 nm (five CdTe atomic monolayers) and that nanowires thinner than three atomic monolayers (0.7 nm) are not sufficiently stable, only three different MSNW species differing by one atomic monolayer are possible. The relative population of each CdTe MSNW species is thus determined by the maximum area of densely packed RNH_2_ monolayers that can assemble at the surface of the nanowires, which is directly related to [RNH_2_] in the reaction medium.

**Figure 7:**
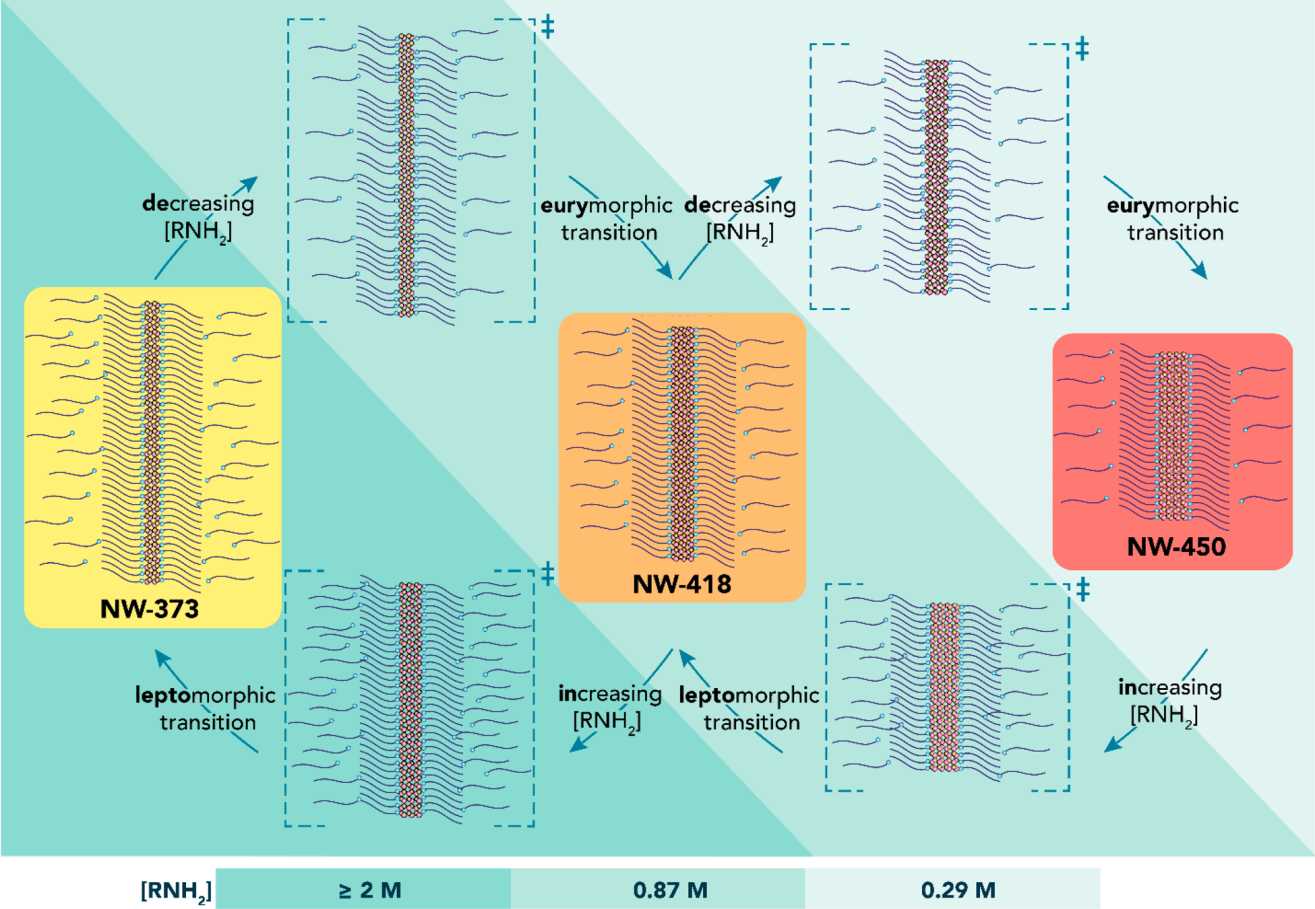
Schematic illustration of the mechanism proposed for the interconversion between different species of CdTe MSNWs in response to changes in the concentration of primary alkylamines (RNH_2_). The three different MSNWs (center panels) are observed as single-species only at specific [RNH_2_]: 0.29 M, 0.87 M and ≥2 M for NW-450 (5 monolayers thick, 1.1 ± 0.2 nm diameter), NW-418 (4 monolayers thick, 0.9 ± 0.2 nm diameter) and NW-373 (3 monolayers thick, 0.7 ± 0.1 nm diameter), respectively. The dashed lines enclose MSNW species that are under unstable conditions (i.e., in a transition state). The responsiveness of the MSNWs to the [RNH_2_] originates from the highly dynamic nature of the RNH_2_ monolayer. At lower [RNH_2_] (top panels), a fully packed ligand monolayer can no longer be sustained at the surface of the thinner MSNWs, resulting in incomplete monolayers. This induces an eurymorphic (from thinner to thicker) magic-diameter interconversion to restore a fully packed ligand monolayer under lower [RNH_2_]. In contrast, the increase of [RNH_2_] (bottom panels) induces a leptomorphic (from thicker to thinner) interconversion because this allows the expansion of the fully packed ligand monolayer at the surface of the MSNWs.

Interestingly, the RNH_2_ monolayer at the surface of the CdTe nanowires is very dynamic because NH_2_ donor heads bind and unbind to the surface of Cd-chalcogenide NCs at fast rates (viz., ≥0.05 ms^–1^).^63,64^ This makes the CdTe MSNWs responsive to changes in [RNH_2_] (Figure 7). Reduction of [RNH_2_] in a solution containing thinner MSNWs will lower the coverage density of the RNH_2_ surface monolayer, making the nanowires less stable and inducing an eurymorphic (from thinner to thicker) magic-diameter interconversion in order to restore a fully packed ligand monolayer through reduction of the total CdTe surface area. This transition is driven by the free-energy gain that originates from the combined effects of restoration of fully packed RNH_2_ monolayers, decrease of the surface free energy of the CdTe nanowire (both through passivation with NH_2_ donor groups and reduction of the surface to volume ratio), and formation of additional Cd–Te bonds (by transfer of CdTe units from surface to interior). In contrast, an increase of [RNH_2_] in a solution containing thicker MSNWs will induce a leptomorphic (from thicker to thinner) magic-diameter interconversion driven by the free-energy gain that originates from the formation of a larger area of fully packed RNH_2_ monolayers.

The leptomorphic transition is slower than the eurymorphic counterpart because it has a higher activation energy since it requires breaking Cd–Te bonds as CdTe units move from the interior to the surface to expand the CdTe surface area available for binding RNH_2_ molecules. The fact that the leptomorphic interconversion is nevertheless observed implies that the free-energy gain from the formation of densely packed RNH_2_ monolayers at the surface of the ultrathin CdTe nanowires largely compensates the energy losses due to breaking of Cd–Te bonds. A possible mechanism for this transformation is the RNH_2_-assisted stepwise transport of CdTe units from the edges to the center of the end facets of the nanowires, thereby simultaneously increasing the length of the nanowire and decreasing its diameter at the edge, while leaving an exposed CdTe surface and a step with respect to the remaining of the nanowire. The tips of the nanowires consist of the polar (002) facets, which have a higher free energy and a lower ligand coverage than the nonpolar lateral facets.^3^ CdTe units at the edges of these facets are thus expected to become highly mobile upon binding to multiple RNH_2_ molecules, drifting from the edge to the center of the (002) facet, where they gain additional stability by forming one additional CdTe bond. The process is also driven by the expansion of the RNH_2_ monolayer since at high [RNH_2_] both the exposed and the newly created Cd sites will bind to RNH_2_ molecules. Importantly, at high [RNH_2_], this process is self-propagating because the CdTe units at the created step have a higher free energy and will thus also be highly mobile upon binding of multiple RNH_2_ molecules, allowing the transport of CdTe units from the side facets to the tips to continue, gradually decreasing the diameter of the nanowire while increasing its length and expanding the area of the RNH_2_ monolayer, until eventually the entire nanowire is converted into the thinnest possible MSNW (NW-373).

We propose that the total volume of the CdTe MSNWs is preserved by the interconversion so that eurymorphic transitions are accompanied by shortening of the nanowires, while leptomorphic transitions lead to longer MSNWs. This bears similarities with the structural reconstruction previously observed during the conversion of template tetragonal umangite Cu_2-*x*_Se nanosheets to hexagonal wurtzite CuInSe_2_ nanosheets by Cu^+^ for In^3+^ cation exchange, through which the lateral dimensions increased at the expense of a reduction in thickness so that the area of the top and bottom (002) polar facets was increased while preserving the total volume.^65^ The driving force for this internal reconstruction process was attributed to the minimization of both the total surface free energy (by maximizing the area of densely packed ligand monolayers) and the reconstruction strain during the structural reorganization process, while keeping the total volume expansion work to a minimum.^65^ The mechanism depicted in Figure 7 also explains the eurymorphic interconversions that occur during the formation and growth of the CdTe MSNWs at [RNH_2_] < 2 M. As discussed above, CdTe monomers likely consist of [(RNH_2_)_*n*_(CdTe)] units, allowing RNH_2_ molecules to be transported to the growing MSNWs and incorporated as capping ligands at the lateral surfaces (section 3.4). This implies that the local, transient [RNH_2_] at the early stages of the reaction is always sufficiently high to promote the initial formation of NW-373. As the nucleation and 1D growth of the NW-373 proceed, the fraction of RNH_2_ molecules that becomes incorporated in the ligand monolayer increases, decreasing the availability of RNH_2_ molecules in solution. Given the dynamic nature of the RNH_2_ monolayer, if [RNH_2_] is insufficient to sustain fully packed RNH_2_ monolayers, the growing MSNWs will interconvert to thicker diameters that can accommodate fully packed monolayers under lower [RNH_2_].

## 4 Conclusions

In this work, we report the room temperature synthesis of three species of micrometer-long ultrathin CdTe magic-size nanowires (MSNWs). For convenience, we refer to them according to the spectral position of their lowest energy absorption transitions: NW-373, NW-, and NW-450 for the MSNWs with the lowest energy exciton transition at 373, 418, and 450 nm, respectively (diameters: 0.7 ± 0.1 nm, 0.9 ± 0.2 nm, and 1.1 ± 0.2 nm, respectively). The MSNWs are obtained from Cd(oleate)_2_ and TOP-Te, provided diphenylphosphine (DPP) and a primary alkylamine (RNH_2_) are present at sufficiently high concentrations (≥3.34 mM and ≥0.1 M, respectively). The population of each MSNW species is determined entirely by the RNH_2_ concentration [RNH_2_] so that single species are only obtained at specific concentrations (viz., 0.29, 0.87, and ≥2.0 M, for NW-450, NW-418, and NW-373, respectively), while mixtures of two different MSNW species are obtained at concentrations intermediate between the specific ones. At [RNH_2_] lower than 2 M, formation of MSNWs is accompanied by quantized growth, which is equivalent to interconversion between MSNW species. Further, the MSNWs remain responsive to [RNH_2_], interconverting from thinner to thicker (eurymorphic transition) upon [RNH_2_] decrease and from thicker to thinner (leptomorphic transition) upon [RNH_2_] increase, with the latter being much slower than the former.

Our results allow us to propose a mechanism for the formation of the CdTe MSNWs and demonstrate that primary alkylamines play crucial roles in all four elementary kinetic steps (viz., monomer formation, nucleation, growth in length, and interconversion between species). The first step in the monomer formation mechanism involves the reaction between DPP-Te and RNH_2_, forming negatively charged Te species that in combination with Cd^2+^ in cadmium oleate favor the formation of MSCs that act as nonclassical nuclei for NW-373, which are always the first to form regardless of [RNH_2_]. Our observations imply that in the presence of primary alkylamines CdTe nucleation and growth follow fundamentally different paths with respect to those available in their absence, suggesting that primary alkylamines impose prohibitively high energy barriers to nucleation and growth of 0D CdTe NCs while favoring the formation of anisotropic 1D NCs. The 1D-directive effect of primary alkylamines is attributed to a synergistic and dynamic interaction between the growing CdTe MSNWs and RNH_2_ molecules bound to both the surface of the MSNWs and to the (CdTe) monomers so that self-assembly of a dense RNH_2_ monolayer provides a significant contribution to the free-energy gain during the growth. The CdTe MSNWs can thus be seen as hybrid organic–inorganic nanostructures, and therefore their diameters are dictated by the balance between two opposing trends: maximization of the area of fully packed RNH_2_ monolayers, which favors the thinnest MSNWs (NW-373) and minimization of the surface to volume ratio of the CdTe nanowires, which favors the thickest MSNWs (NW-450). The RNH_2_ monolayer is however very dynamic, making the MSNWs responsive to changes in [RNH_2_]. Reduction of [RNH_2_] in a solution containing thinner MSNWs lowers the coverage density of the RNH_2_ monolayer, inducing an eurymorphic magic-diameter interconversion to restore a fully packed ligand monolayer through reduction of the total CdTe surface area. In contrast, increase of [RNH_2_] in a solution containing thicker MSNWs induces a leptomorphic magic-diameter interconversion driven by the free-energy gain that originates from the formation of a larger area of fully packed RNH_2_ monolayers. It is thus clear that primary alkylamines are the decisive element in the creation of a reaction pathway that leads exclusively to CdTe MSNWs. The insights provided by our work thus contribute toward unravelling the mechanisms behind the formation of shape-controlled and atomically precise magic-size semiconductor nanostructures.

## Supporting Information Available

Cryo-TEM images of NW-373 and NW-418 MSNWs. TEM images illustrating e-beam induced damage of MSNWs. TEM images and absorption spectra of the CdTe products obtained with different primary alkylamines and with tertiary and secondary alkylamines. Absorption spectra showing temporal evolution for different [DDA] and different [DPP]. Temporal evolution of the absorbance at 300 nm for different [DDA]. Reaction mechanism for the formation of CdSe MSCs from Cd-benzoate and DPP-Se. Absorption spectra of products obtained with superhydride instead of DPP. Absorption spectra and TEM images of products obtained at 120 °C. Detailed description of the procedure used to quantitatively analyze the absorption spectra and fit them as a linear combination of the spectra of single-species MSNWs, as well as fitted spectra for several [DDA] and time points. Absorption spectra showing interconversion between different MSNW species (PDF)
