# Supplementary material for: Room-Temperature Interconversion Between Ultrathin CdTe Magic-Size Nanowires Induced by Ligand Shell Dynamics
Source: J Phys Chem C Nanomater Interfaces. 2022 Aug 31;126(36):15280–97. doi: 10.1021/acs.jpcc.2c04113 (PMC9483966; doi:10.1021/acs.jpcc.2c04113)
Supplement: Supplementary file 1 — jp2c04113_si_001.pdf [file jp2c04113_si_001.pdf]

Supporting Information

Room-Temperature Interconversion Between Ultrathin CdTe Magic-Size Nanowires Induced by Ligand Shell Dynamics

*Serena Busatto,[a] Claudia Spallacci,[a] Johannes D. Meeldijk,[b] Stuart Howes,[c] and Celso de Mello Donega[a,]**

a. Condensed Matter and Interfaces, Debye Institute for Nanomaterials Science, Utrecht University, 3508 TA Utrecht, The Netherlands

b. Materials Chemistry and Catalysis, Debye Institute for Nanomaterials Science, Utrecht University, 3508 TA Utrecht, The Netherlands

c. Structural Biochemistry, Bijvoet Centre for Biomolecular Research, Utrecht University, Padualaan 8, 3584 CH Utrecht, The Netherlands.

***Corresponding Author: c.demello-donega@uu.nl**

Section S1. Transmission Electron Microscopy Images (Figures S1 to S3)

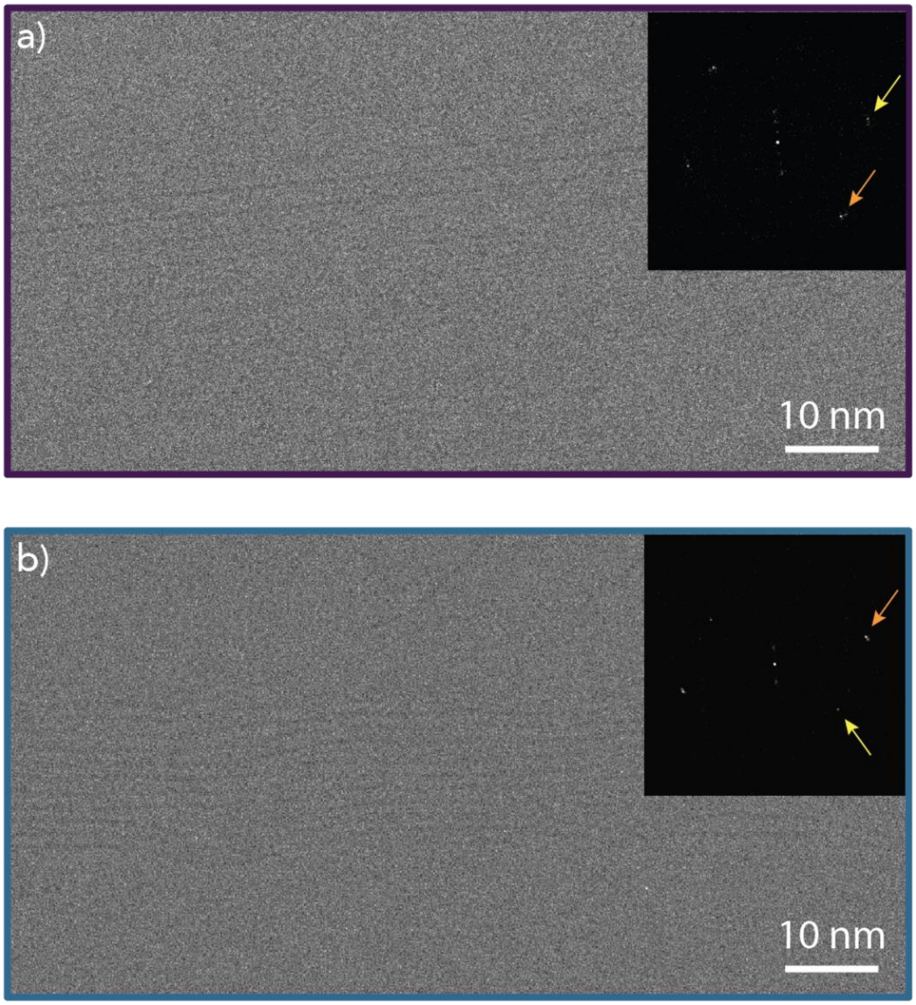

Figure S1. Low e-dose cryo-TEM images of the CdTe products obtained with **(a)** 2.04 M DDA (purple curve in Figure 1a of the main text, first absorption peak at 373 nm) and **(b)** 0.87 M DDA (blue curve in Figure 1a of the main text, first absorption peak at 418 nm). The fast Fourier transform (FFT) patterns of the images are given in the respective insets. The yellow and orange arrows indicate, respectively, interplanar spacings of 4.1 Å and 3.8 Å in panel (a) and 4.3 Å and 3.6 Å in panel (b). The average diameter of the nanowires obtained from ~20 measurements from different cryo-TEM images is 0.7 ± 0.1 nm and 0.9 ± 0.2 nm for the samples shown in (a) and (b), respectively. The use of very low e-doses, which is responsible for the poor contrast of the images, proved essential to preserve the integrity of the nanowires (at higher e-doses no diffraction spots are observed in the FFT patterns).

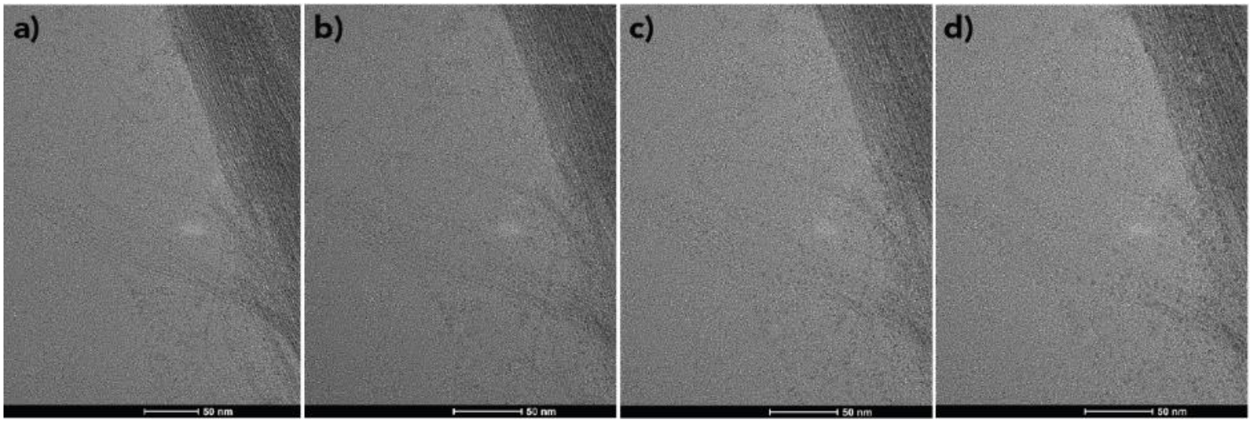

Figure S2. Sequential TEM images of the CdTe product obtained with 0.29 M DDA (green curve in Figure 1a of the main text, first absorption peak at 450 nm), acquired every 10 seconds over the same area of the TEM grid (exposure time is 1 s). It is evident that the nanowires suffer from severe and fast e-beam damage.

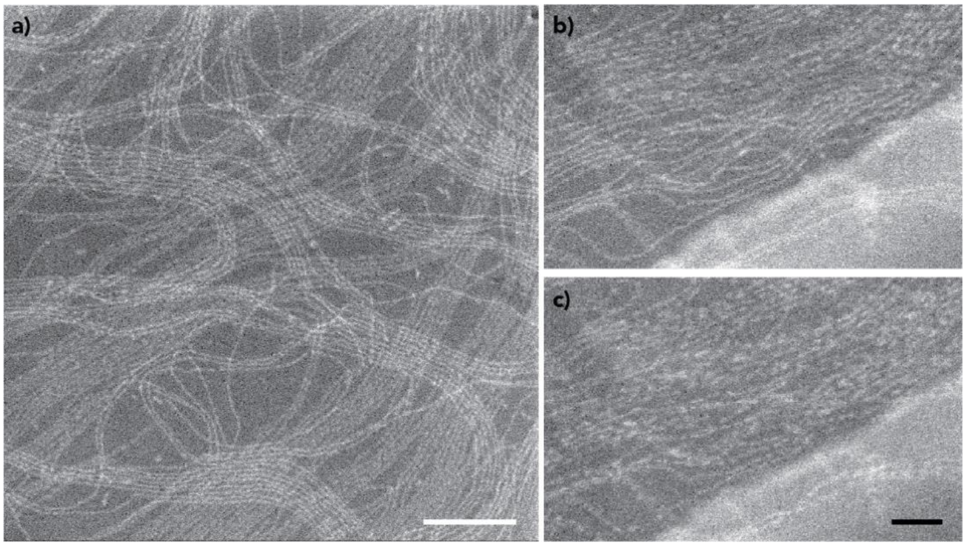

Figure S3. (a) Cryo-STEM image of the same sample shown in Figure S1. **(b, c)** Sequential cryo-STEM images taken on the same area with a time interval of ~10 s. Scale bar is 20 nm.

Section S2. Reaction mechanism: influence of the nature and concentration of the alkylamine and alkylphosphine (Figures S4 to S14)

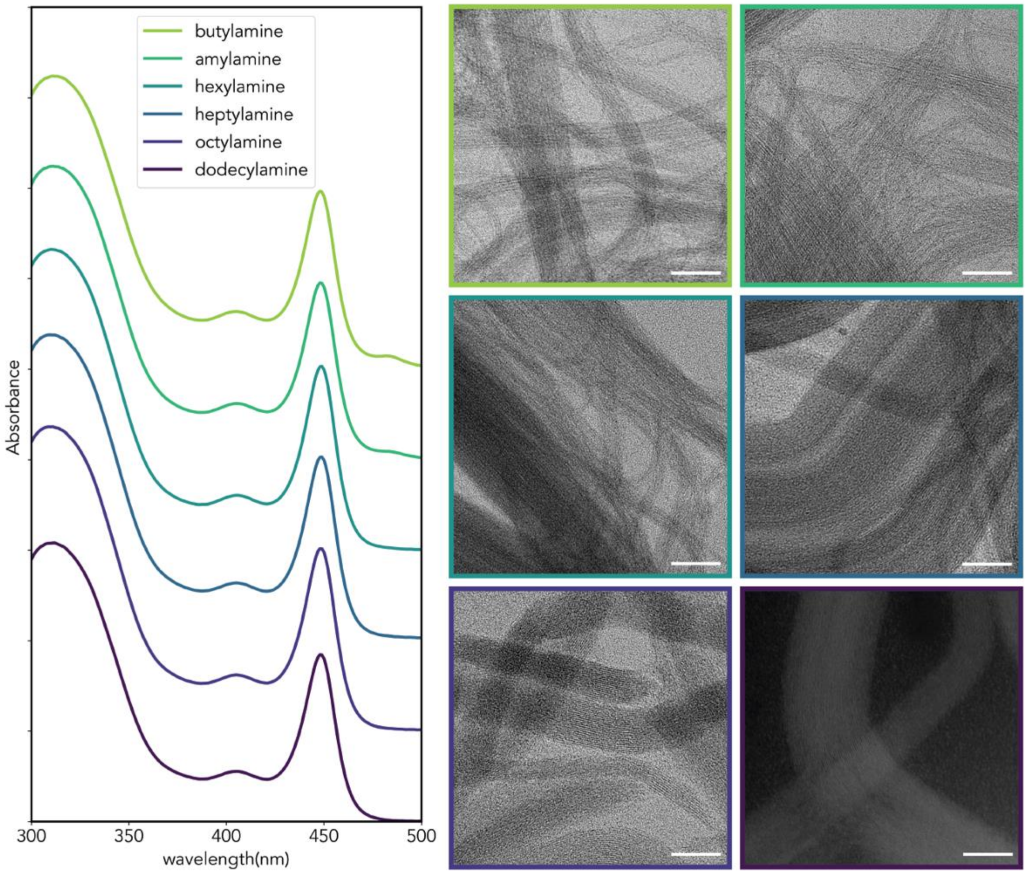

Figure S4. Absorption spectra (left) and TEM images (right) of the CdTe products obtained after 45 min of reaction for different primary alkylamines (0.2 M). The color code is the same in both sets of panels. All other reaction variables and dilution factors are the same. The bottom right panel is a HAADF-STEM image, while the others are Bright Field TEM images. Scale bar is 50 nm in all cases. Magic-size nanowires with the lowest energy absorption transition at 450 nm (NW-450, 1.1 nm diameter) are obtained in all cases, regardless of the chain length. The only difference between the different primary alkylamines is that the colloidal stability decreases for chains shorter than 8 carbons leading to an increase in turbidity over time accompanied by the appearance of a weak peak at 483 nm (see spectra for amylamine and butylamine). This additional peak is attributed to ultrathin nanoribbons formed by lateral fusion of nanowires (see Figure S14b below for a representative absorption spectrum), likely due to the less effective steric stabilization offered by the shorter chain alkylamines.

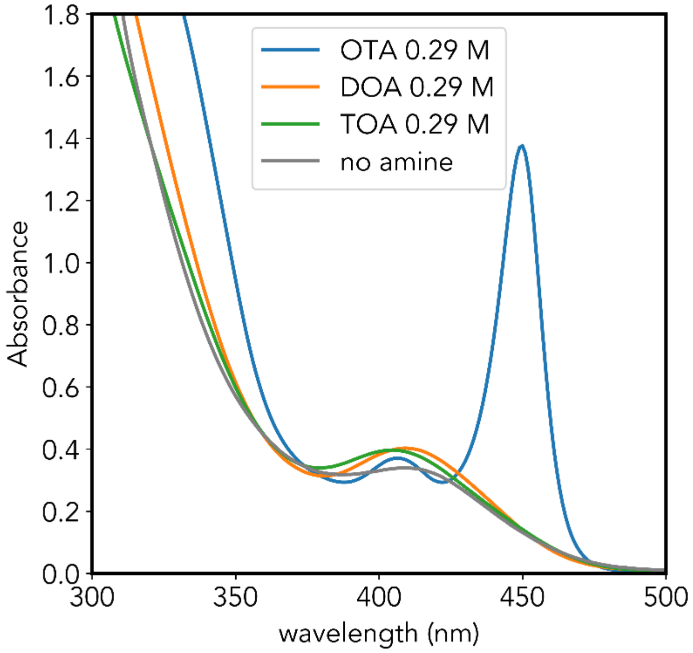

Figure S5. Absorption spectra of the CdTe products obtained after 48 h of reaction for the same concentration (0.29 M) of three different amines: *n*-octylamine (OTA, a primary alkylamine), di-*n*-octylamine (DOA, a secondary alkylamine), and tri-*n*-octylamine (TOA, a tertiary alkylamine). The absorption spectrum of the product obtained without the addition of an alkylamine is also shown for comparison. All other reaction variables and dilution factors are the same.

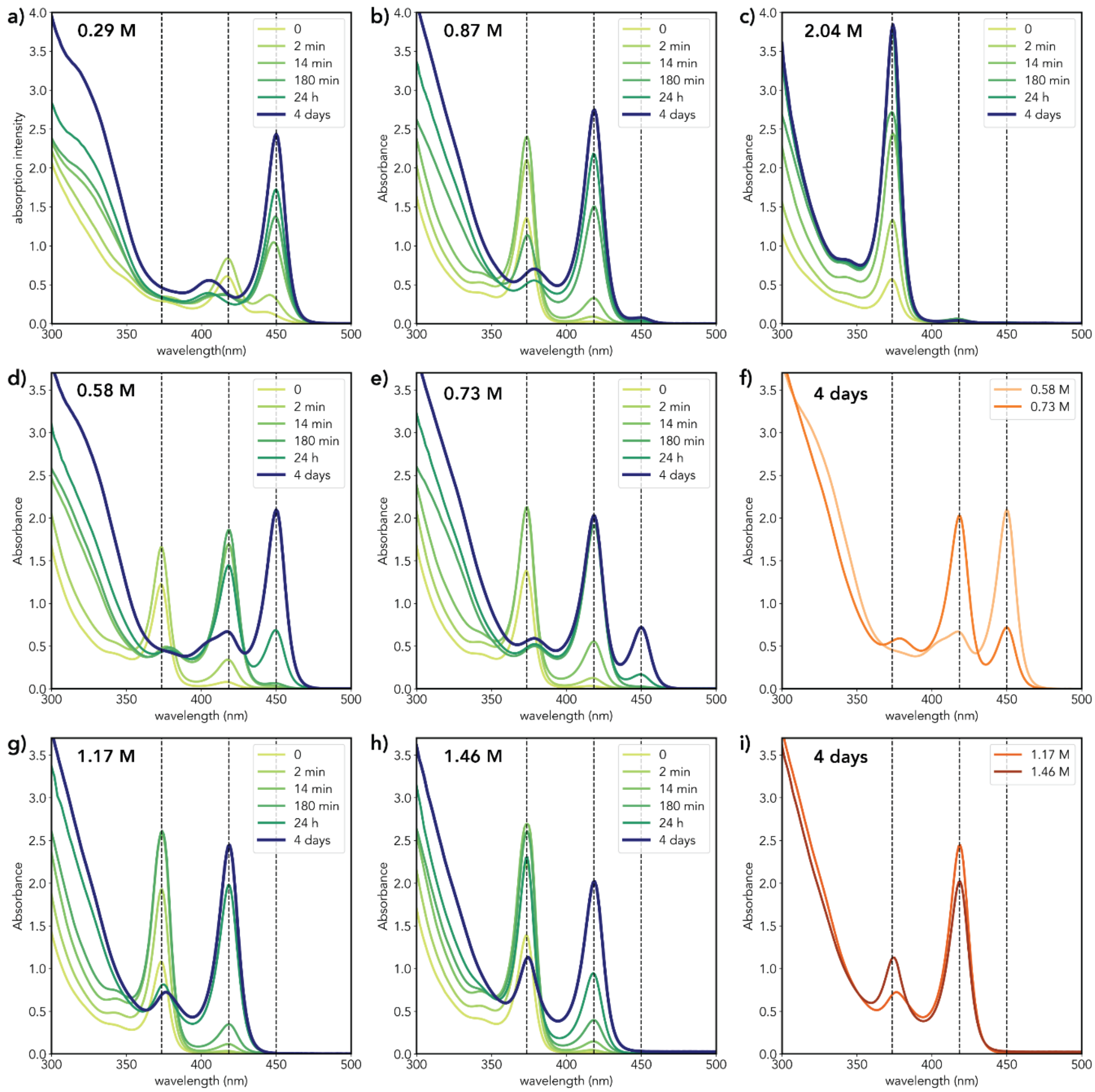

Figure S6. Temporal evolution of the absorption spectra of the CdTe products obtained for different DDA concentrations. All other reaction variables are the same ([DPP]= 33 mM). The spectra were acquired in cuvettes with 1-mm optical path using the neat reaction mixture. Vertical dashed lines indicate the lowest energy absorption peak of each species of magic-size nanowires (373 nm, 418 nm, and 450 nm, respectively). The first measurement (indicated by “0” in the legends) was carried out 30 s after the preparation of the reaction mixture. Panels (a-c) show the concentrations that yield single nanowire species after 24 h. Panels (f) and (i) show the absorption spectra after 4 days for DDA concentrations that yield mixed species.

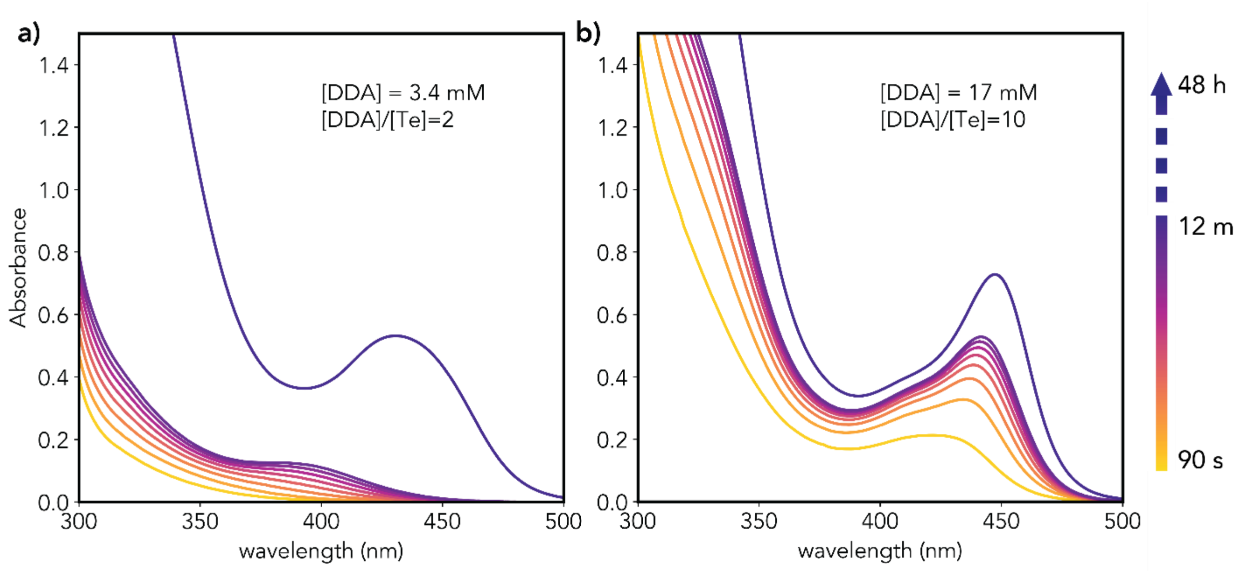

Figure S7. Temporal evolution of the absorption spectra of the CdTe products obtained for two different concentrations of dodecylamine (DDA) indicated in the legend. The molar ratio between DDA and Tellurium (the limiting reagent, present as TOP-Te) is indicated as [DDA]/[Te]. All other reaction variables and the dilution factor of the reaction mixture are the same. The spectra from 90 s (yellow) to 12 min (purple) were acquired at intervals of 1.5 minutes after the start of the reaction, while the last spectrum (violet) was acquired after 48 h.

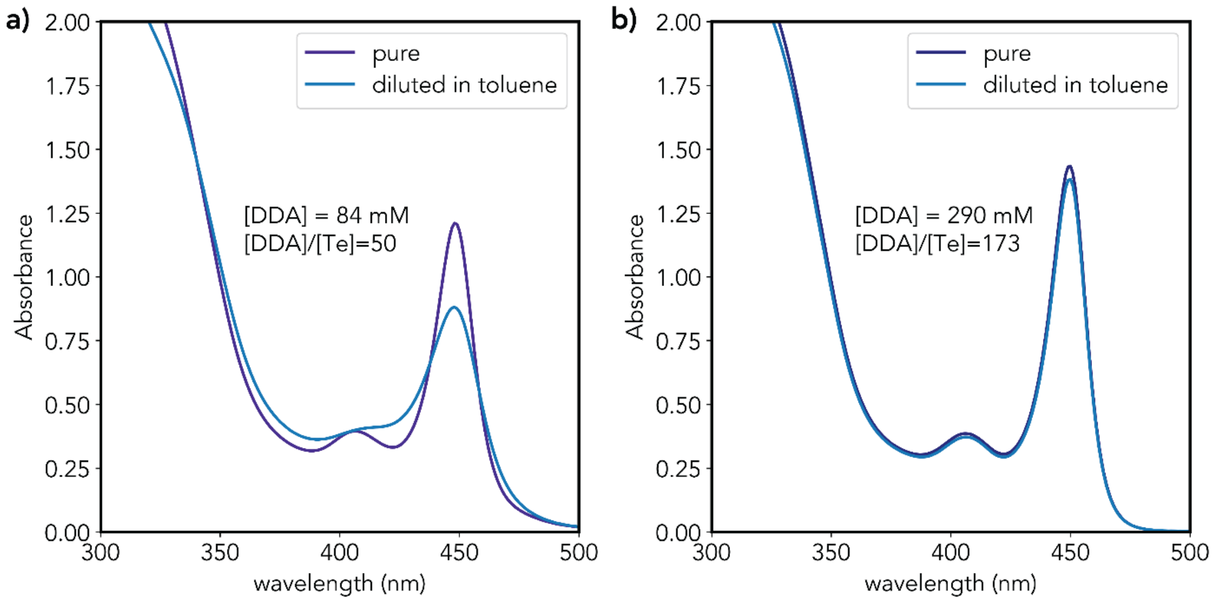

Figure S8. Absorption spectra of the CdTe products obtained after 48 h of reaction for two different DDA concentrations (indicated as [DDA] in the legend) prior to ("pure") and after a factor 10 dilution in toluene. The molar ratio between DDA and Tellurium (the limiting reagent, present as TOP-Te) is indicated as [DDA]/[Te]. All other reaction parameters were the same. The pure reaction mixture was measured in a cuvette with 1 mm optical path length, while the diluted solution was measured in a cuvette with 10 mm optical path length. In this way the absorbance of the neat and diluted solutions would be the same in the absence of transformations induced by dilution. This is the case for the 290 mM DDA but not for the 84 mM DDA solution, showing that a DDA concentration of 8.4 mM is insufficient to preserve the integrity of the NW-450.

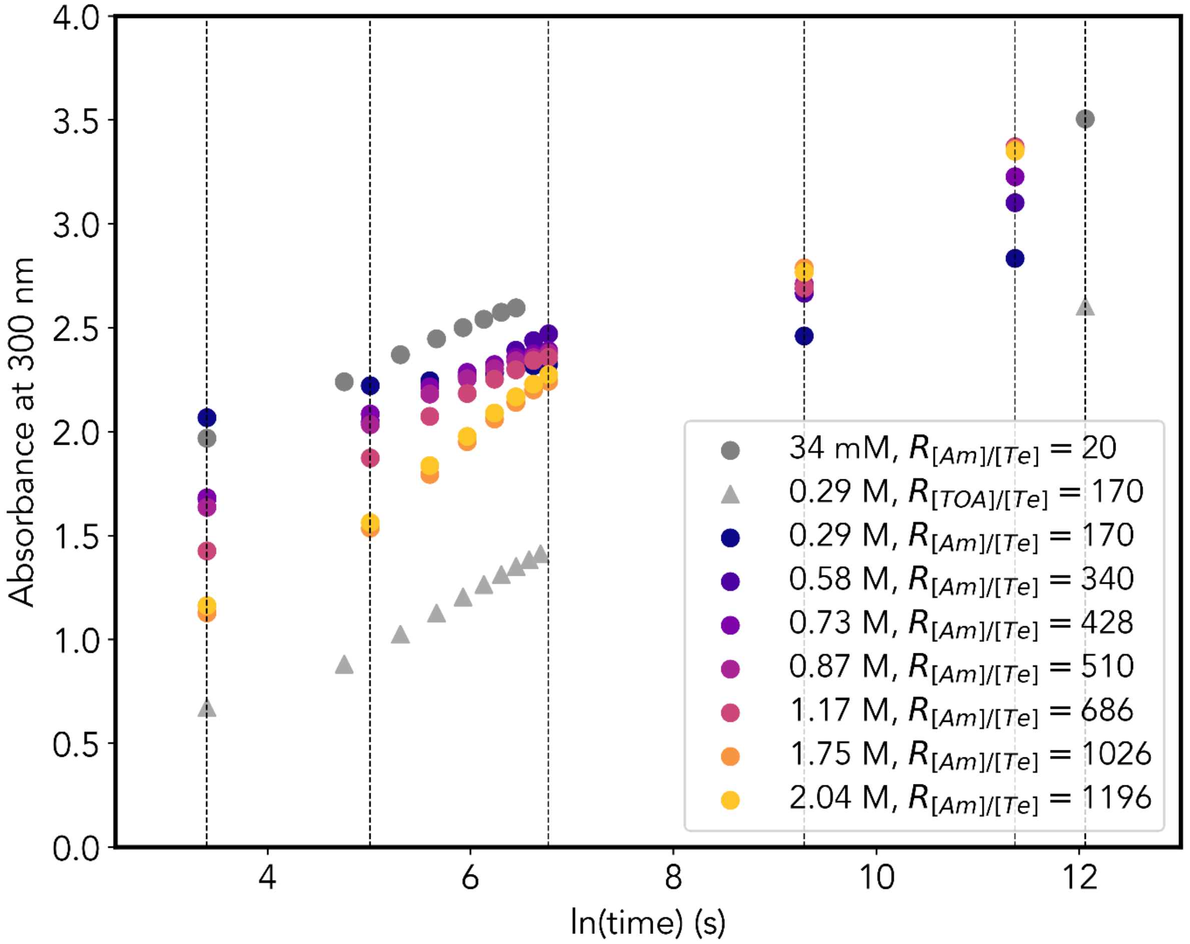

Figure S9. Temporal evolution of the absorbance at 300 nm of the CdTe products obtained for 0.29 M trioctylamine (TOA) and for different concentrations of dodecylamine (DDA) indicated in the legend (*note that time is given on a logarithmic scale*). All other reaction variables and the dilution factor of the reaction mixture are the same ([DPP]= 33 mM). $R_{[Am]/[Te]}$ gives the molar ratio between DDA and Tellurium (the limiting reagent, present as TOP-Te). $R_{[TOA]/[Te]}$ gives the molar ratio between TOA and Tellurium. The absorbance values were obtained from spectra acquired in cuvettes with 1-mm optical path using the neat reaction mixture. The dashed lines mark the following times: 30 s, 2 min, 14 min, 3 h, 24 h and 48 h, respectively.

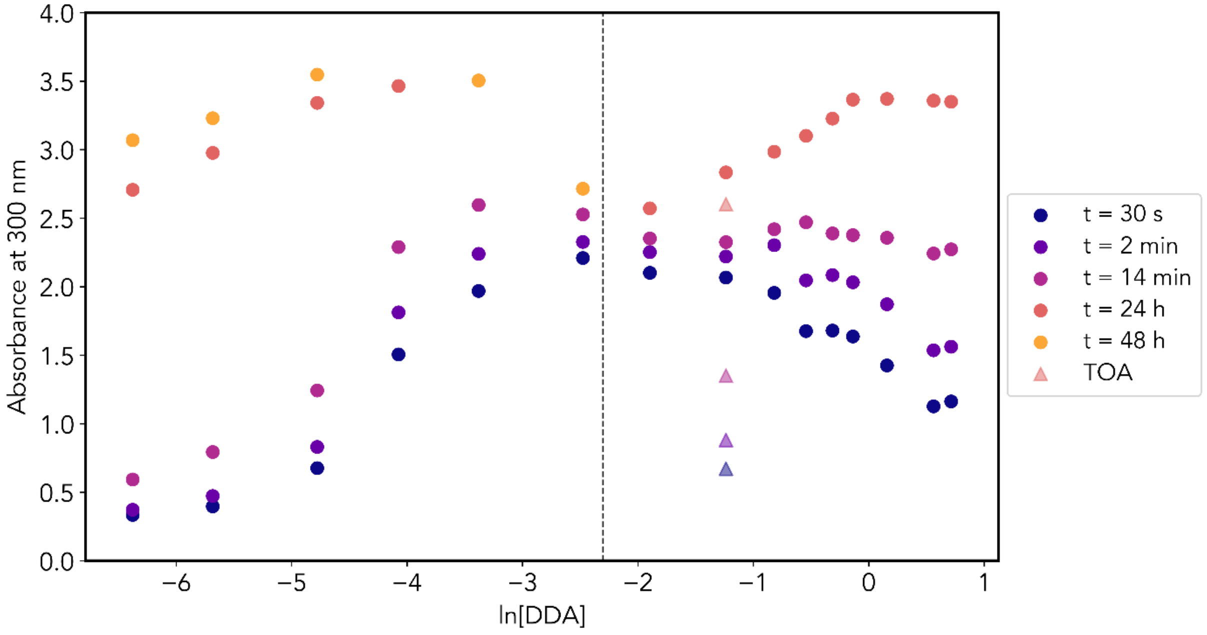

Figure S10. Absorbance at 300 nm of the CdTe products obtained after selected reaction times for 15 different DDA concentrations (from 1.7 mM to 2.04 M, given on a logarithmic scale). For comparison, the absorbance at 300 nm of the CdTe products obtained at the same reaction times for a trioctylamine (TOA) concentration of 0.29 M is also shown (triangles). The vertical dashed line marks the critical concentration for the formation of nanowires (0.1 M).

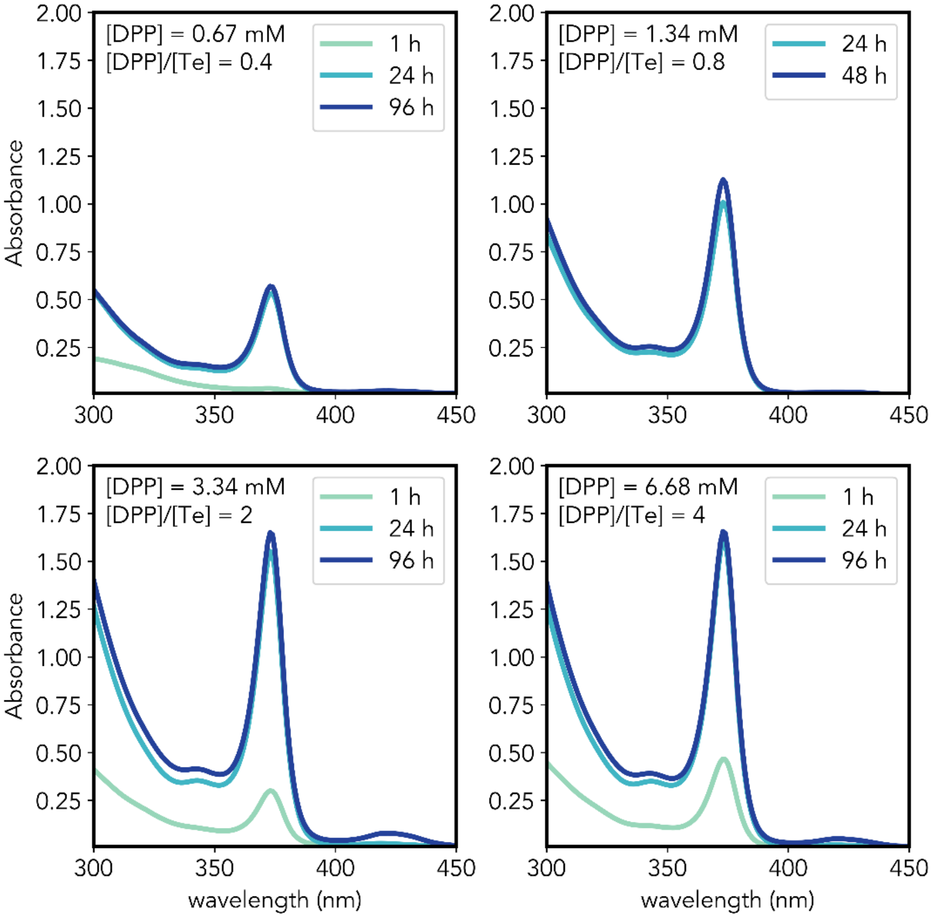

Figure S11. Temporal evolution of the absorption spectra of the CdTe products obtained for 2.04 M DDA and four different DPP concentrations. The concentration of Te is the same in all cases.

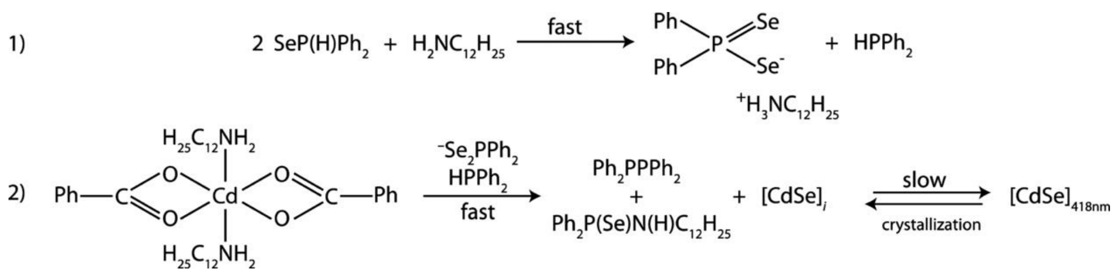

Figure S12. Reaction pathway for the formation of CdSe Magic-Size Clusters from Cadmium Benzoate and Diphenylphosphine Selenide. In the first step, diphenylphosphine selenide reacts with primary alkylamine to give the diselenophosphinate anion. This anion subsequently reacts (in the presence of secondary phosphine and primary alkylamine) with cadmium benzoate to give organic coproducts Ph_2PPPh_2 and $Ph_2P(Se)N(H)C_{12}H_{25}$ and $[CdSe]_i$, which slowly crystallizes, giving a CdSe magic-size cluster with lowest energy absorption peak at 418 nm (*viz*., $[CdSe]_{418nm}$). Control experiments at ambient temperatures reveal that DPPSe and dodecylamine rapidly and quantitatively convert to $HPPh_2$ and $[Se_2PPh_2]^-$ $[H_3NC_{12}H_{25}]^+$. When the diselenoate/ diphenylphosphine mixture is preformed and added to the reaction mixture to initiate growth, $[CdSe]_{418nm}$ is obtained with the same overall rate and total conversion, indicating that the diphenyldiselenophosphinate is a kinetically competent precursor. Reproduced from: B. M. Cossairt and J. S. Owen, *Chem. Mater*. **2011**, *23*, 3114–3119.

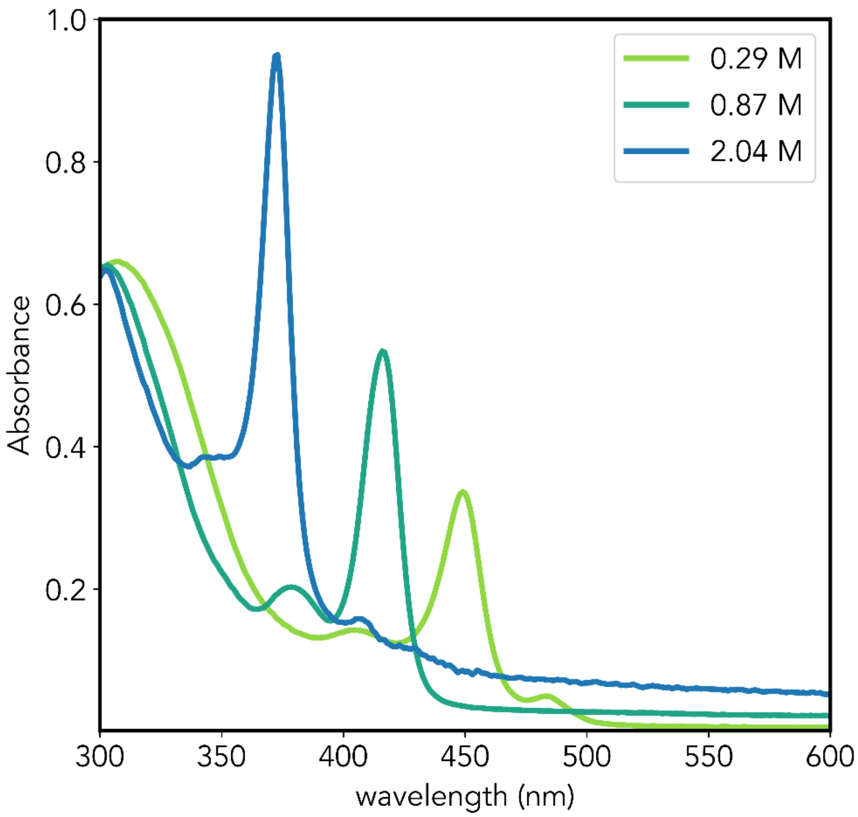

Figure S13. Absorption spectra of the CdTe products obtained after 48 h of reaction for different DDA concentrations and replacing DPP by Superhydride (20 mM). All other reaction variables and the dilution factor of the reaction mixture are the same.

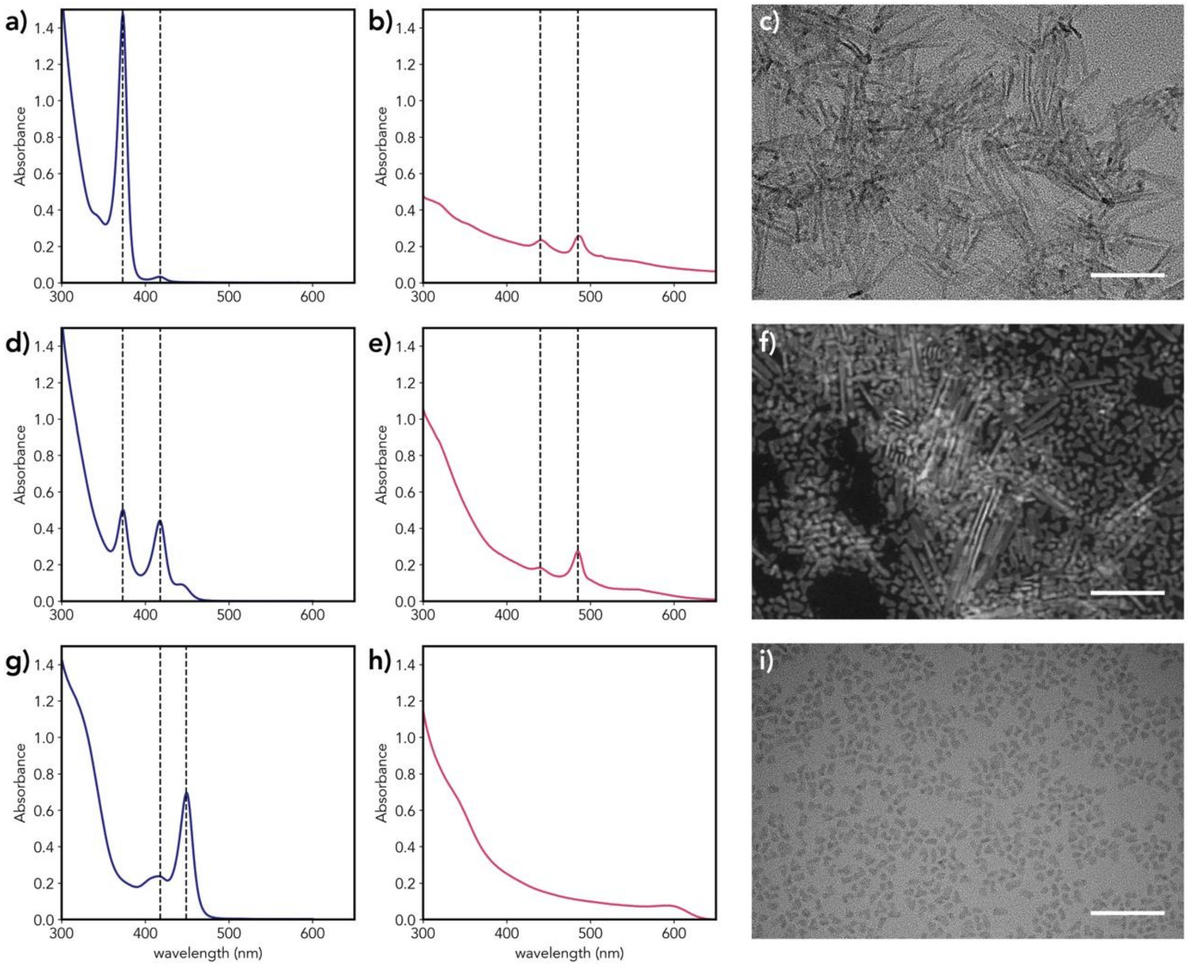

Figure S14. **(a,b)** Absorption spectra of the CdTe products obtained for [DDA] = 2.04 M, after 3 h at room temperature **(a)**, followed by 24 h at 120 °C **(b)**. **(c)** Bright field TEM image of the reaction product obtained after 24 h at 120 °C and yielding the absorption spectrum shown in (b). **(d, e)** Absorption spectra of the CdTe products obtained for [DDA] = 0.87 M after 3 h at room temperature **(d)**, followed by 24 h at 120 °C **(e)**. **(f)** HAADF-STEM image of the reaction product obtained after 24 h at 120 °C and yielding the absorption spectrum shown in (e). **(g, h)** Absorption spectra of the CdTe products obtained for [DDA] = 0.29 M after 3 h at room temperature **(g)**, followed by 24 h at 120 °C **(h)**. **(i)** Bright field TEM image of the reaction product obtained after 24 h at 120 °C and yielding the absorption spectrum shown in (h). The scale bar in all (S)TEM images corresponds to 50 nm.

Section S3. Quantitative analysis of the absorption spectra of the reaction mixtures (Figures S15 to S26).

S3.1. Optical path lengths and dilution factors

The absorption spectra investigated in this work were measured using three different concentrations, depending on the purpose of the measurement (Table S1). To follow the temporal evolution of the absorption spectra *in-situ*, without any dilution, quartz cuvettes with 1 mm optical path length were used. Experiments that required dilution (*e.g.*, to study the impact of the concentration of alkylamines on the nature and stability of the MSNW species) employed quartz cuvettes with 10 mm optical path length and either a factor 10 or 16.7 dilution (dilution factors 0.1 and 0.06, respectively, Table S1).

To allow a direct quantitative comparison between spectra measured for the neat reaction mixture in 1 mm cuvettes and for diluted solutions in 10 mm cuvettes a scaling relation must be used. Knowing that $\mathbf{A} = \varepsilon \times C \times \ell$ one can write: $\mathbf{A}_{\ell=10} = \varepsilon \times C_d \times \mathbf{10}$ and $\mathbf{A}_{\ell=1} = \varepsilon \times C_0 \times \mathbf{1}$. It then follows that $(\mathbf{A}_{\ell=10}/\mathbf{A}_{\ell=1}) = (C_d/C_0) \times (\mathbf{10/1}) = (\mathbf{DF}) \times \mathbf{10}$ and $\mathbf{A}_{\ell=10} = \mathbf{A}_{\ell=1} \times (\mathbf{DF}) \times \mathbf{10}$

The preferred dilution factor was 0.1 because then the absorbance was the same for both the neat and diluted samples. However, the optical density of solutions with DF= 0.1 was often too high, leading to saturation effects in the measurements. For this reason, most of the absorption spectra acquired in 10 mm cuvettes used solutions with DF= 0.06, unless otherwise specified.

Table S1. Optical path lengths and dilution factors used for the absorption spectra measurements (ℓ= optical path length, DF= Dilution Factor, C_0= concentration of the neat reaction mixture, C_d= concentration after dilution).

**ℓ (mm)**	**Total Volume (mL)**	**Sample Volume (mL)**	**Toluene Volume (mL)**	**DF (C_d/C_0)**
1	0.300	0.300	0	1
10	2.500	0.250	2.250	0.1
10	2.500	0.150	2.350	0.06

S3.2. Absorption spectra as linear combinations of the absorption spectra of single-species magic-size nanowires

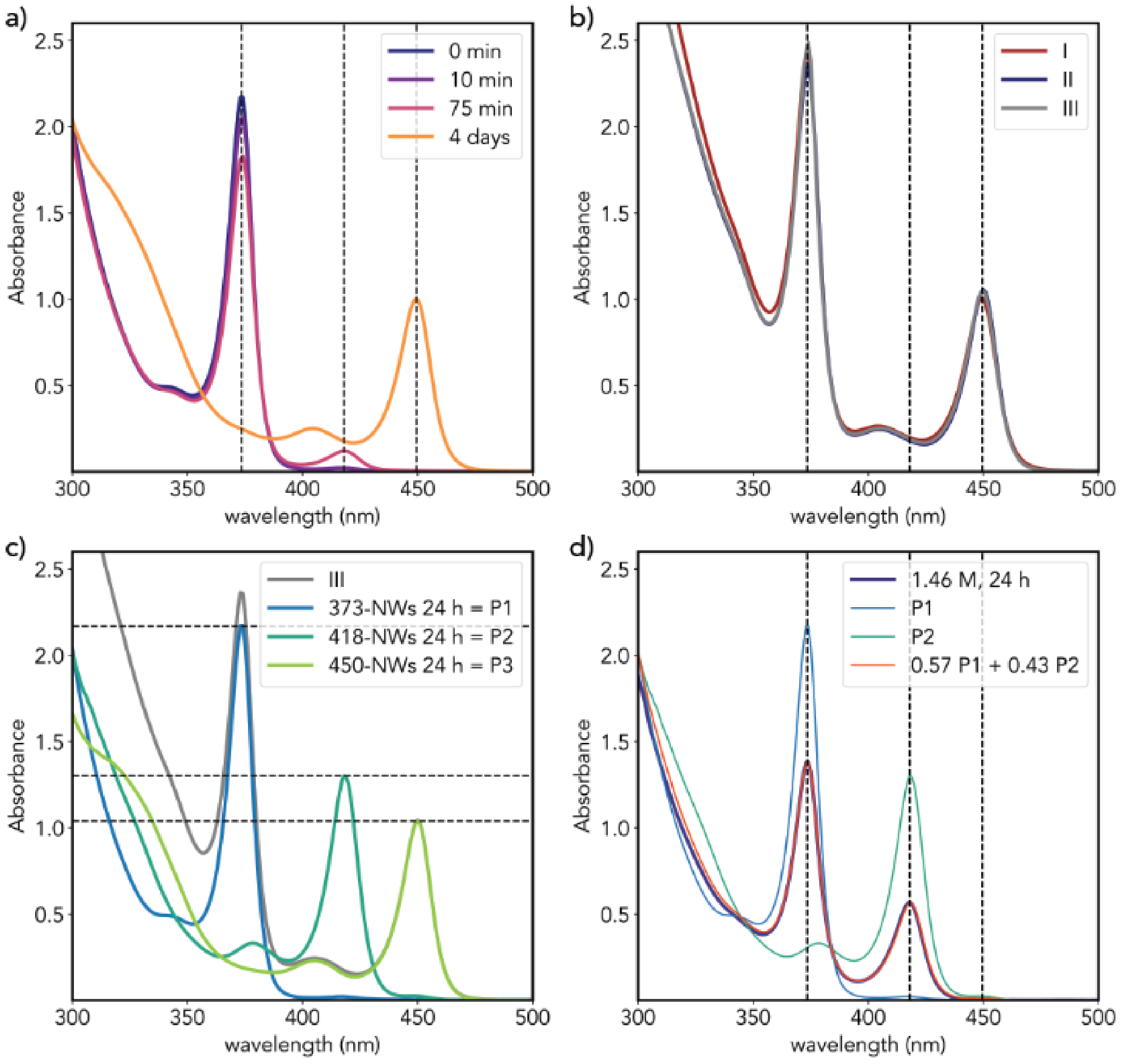

Figure S15. **(a)** Temporal evolution of the absorption spectra of a solution initially containing single-species CdTe NW-373 ([DDA]= 2.04 M in the reaction mixture), after its dilution to a DDA concentration that stabilizes CdTe NW-450 ([DDA]= 0.29 M). The measurements were carried out in a sealed 10-mm quartz cuvette. The first measurement (0 min) was acquired immediately after the dilution. This experiment shows that upon dilution to a DDA concentration of 0.29 M, single-species NW-373 are fully converted to single-species NW-450 after 4 days.

(b) I. Sum of the initial (0 min) and final (4 days) absorption spectra shown in panel (a). **II.** Absorption spectrum of a mixture composed of equal volumes (150 ul) of neat reaction mixture containing single-species NW-373 and NW-450 measured in a sealed 1-mm cuvette immediately after the mixing. The reaction mixtures are the same used to obtain the 24 h spectra shown in, respectively, panels (c) and (a) of Figure S6 above. **III**. Sum of the absorption spectra of single-species NW-373 and NW-450 measured after 24 h of reaction in cuvettes with 1 mm optical path length (the original spectra are shown in panels (c) and (a) of Figure S6 above). Scaling factors were used to account for the differences in absorbance due to different concentrations and optical path lengths. Spectra I and III result from the sum of two independently measured absorption spectra, which is equivalent to doubling the original absorbance values. To allow comparison with spectrum II, spectra I and III must thus be divided by 2. Spectra II and III were measured over the same optical path length (*viz*., 1 mm) and for solutions with the same concentration (*i.e*., neat reaction mixtures), so they can be directly compared to each other without further scaling. However, the original spectra used to obtain spectrum I were measured for diluted solutions in a cuvette with 10 mm optical path length. To allow comparison with spectra II and III, spectra I must thus be additionally divided by 0.6 (see **Table S1** above for details). After the scaling procedure, spectra I, II, and III overlap almost perfectly. This implies that the conversion of the NW-373 into NW-450 is quantitative, without the formation of any other byproduct, and that the initial and final concentrations of CdTe NWs are the same as those obtained by direct synthesis. We can thus conclude that the amount of CdTe formed at reaction times ≥24 h is the same, regardless of the DDA concentration.

(c) The conclusion above (panel b) implies that the absorption spectra of single-species MSNWs, such as those acquired after 24 h for DDA concentrations of 0.29 M (NW-450), 0.87 M (NW-418) and 2.04 M (NW-373), correspond to the same amounts of CdTe. These spectra are shown, respectively, in panels (a), (b), and (c) of Figure S6 above and are reproduced here and labeled P1, P2, and P3, respectively. The horizontal dashed lines mark the absorbance values of the lowest energy peak of each MSNW species. Spectrum III is the same as shown in panel (b) and is equivalent to [P1 + P3].

(d) The analysis carried out in panels (a-c) establishes that absorption spectra acquired after 24 h correspond to the same amount of CdTe regardless of the DDA concentration in the reaction mixture. In other words, the total amount of CdTe distributed over all MSNWs formed in the

reaction mixture after 24 h is the same for all DDA concentrations, regardless of the dimensions of the nanowires. Given that only three different species of magic-size nanowires (MSNWs) can be formed and that each of them is characterized by a unique absorption spectrum, this implies that linear combinations of the spectra labelled P1, P2, P3 in panel (c) can be used to reproduce the absorption spectra of any suspension containing the three MSNW species in any ratio. Most importantly, the coefficients of these linear combinations are proportional to the partition of the total amount of CdTe available in the reaction medium between the different species of MSNWs. Considering that the characteristic absorption spectrum of a MSNW is dictated only by its diameter (the confinement dimension) while the total absorbance is determined by total volume of absorbing material, it follows that the coefficients obtained by fitting the absorption spectra as linear combinations of the spectra of single-species MSNWs are equivalent to the relative volume fraction of each MSNW species. The fitting coefficients thus give the relative amount of CdTe locked into each of the different MSNW species, but provide no information concerning the concentration (*i.e.*, number density) of each species, given that the total absorption cross-section of one longer MSNW with length *n*L is identical to that of *n* shorter MSNWs with length L.

An illustrative example of this procedure is given in panel **(d)**. The absorption spectrum of the CdTe product obtained after 24 h of reaction for 1.46 M DDA (dark blue) can be precisely reproduced by a linear combination (orange) of the P1 and P2 spectra of panel c (blue and green curves, respectively). The coefficients of the linear combination give a measure of the relative volume fraction of each species in solution, which is 57% NW-373 and 43% NW-418 for the reaction carried out under 1.46 M DDA.

S3.3. Temporal evolution of the formation of CdTe MSNWs for different DDA concentrations analyzed by fitting the spectra as linear combinations of spectra of single-species MSNWs (Figures S16 to S23).

Each of the following figures shows the temporal evolution of the absorption spectra of the CdTe products obtained for different DDA concentrations (0.29 M, 0.58 M, 0.73 M, 0.87 M, 1.17 M, 1.46 M, 1.75 M, and 2.04 M). For each DDA concentration six time points were analyzed (t= 0, 2 min, 14 min, 3 hours, 24 hours, 4 days). The t= 0 spectra were collected ~30 s after the mixture of all reagents. The linear combinations (LCs) of the spectra of the three single-species MSNWs (NW-373, NW-418, and NW-450, spectra P1, P2, and P3, in Figure S15c above) that best reproduce the experimental spectra are plotted in orange. The single-species spectra used in each LC are plotted as dashed lines, using the same color code used in Figure S15c. The legend also gives the equation describing the best LC, which is as:

$c1 \times P1 + c2 \times P2 + c3 \times P3$

where the coefficients c1, c2 and c3 are proportional to the relative volume fraction of each MSNW species at a particular time point. The sum of the coefficients (written on each panel) is proportional to the total amount of CdTe formed after a given time interval. It is interesting to notice that the amount of CdTe formed after 24 hours is very similar for all [DDA] (sum of the coefficients ～1 in every sample). After 4 days, the amount of CdTe appears to increase (sum of coefficients >1) in a way that is inversely proportional to the DDA/toluene volume ratio in the sample: it is zero for the sample with [DDA] = 2.04 M (DDA/toluene = 1) and maximum for the sample with [DDA] = 0.29 M (DDA/toluene = 0.14). We thus attribute this apparent increase in CdTe concentration to toluene evaporation, since its boiling point (110.6 °C) is much lower than that of DDA (259 °C), and the cuvettes were sealed but not hermetically tight.

The data extracted from these linear combinations are used to construct the 3D-Histogram presented in Figure 5 in the main text.

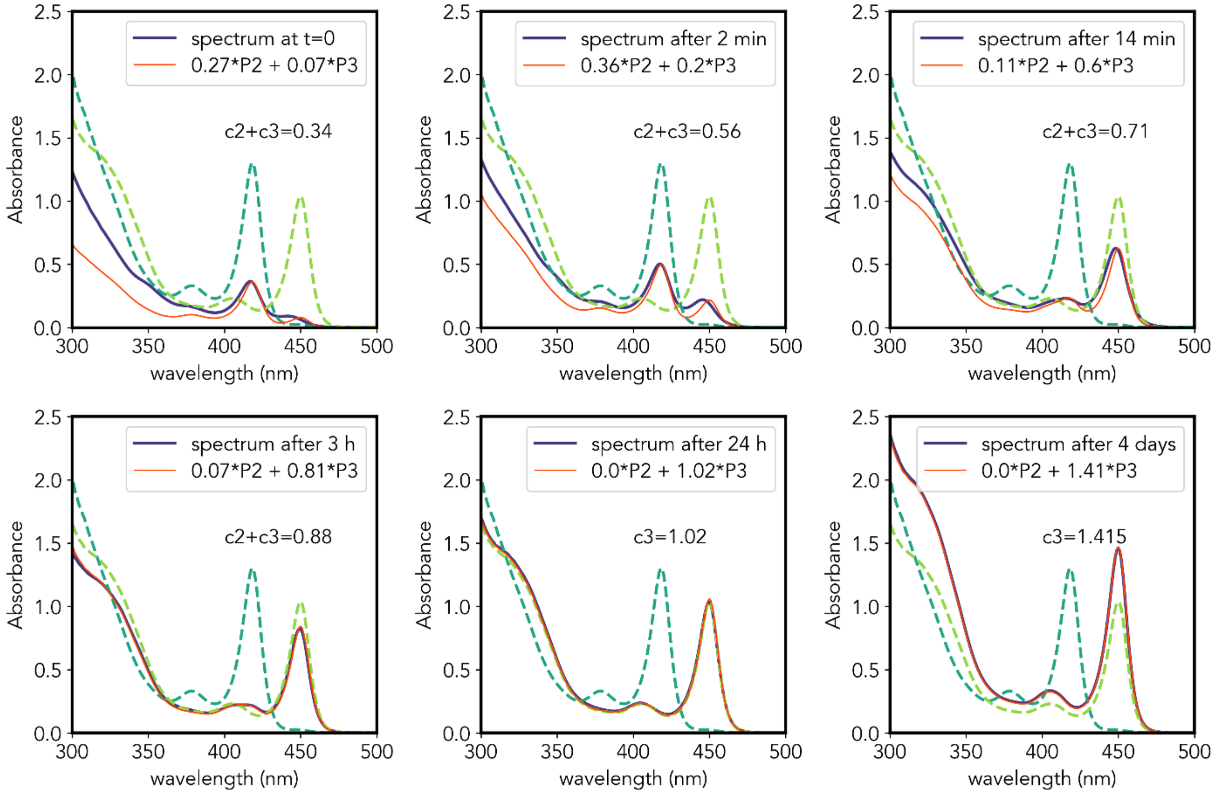

Figure S16. Temporal evolution of the absorption spectra of the CdTe products obtained for **[DDA] = 0.29 M**. Each panel corresponds to one time point, indicated in the legend. The measured spectra are given in blue. The first measurement (indicated by "0") was carried out ~30 s after the preparation of the reaction mixture. The linear combinations (LC) of the spectra of the three single-species MSNWs (NW-373, NW-418, and NW-450, spectra P1, P2, and P3, in Figure S15c above) that best reproduce the experimental spectra are plotted in orange. The single-species spectra used in each LC are plotted as dashed lines, using the same color code used in Figure S15c. The legend also gives the equation describing the best LC and the sum of the relevant coefficients c*N* (*N*= 1, 2, or 3) at each time point.

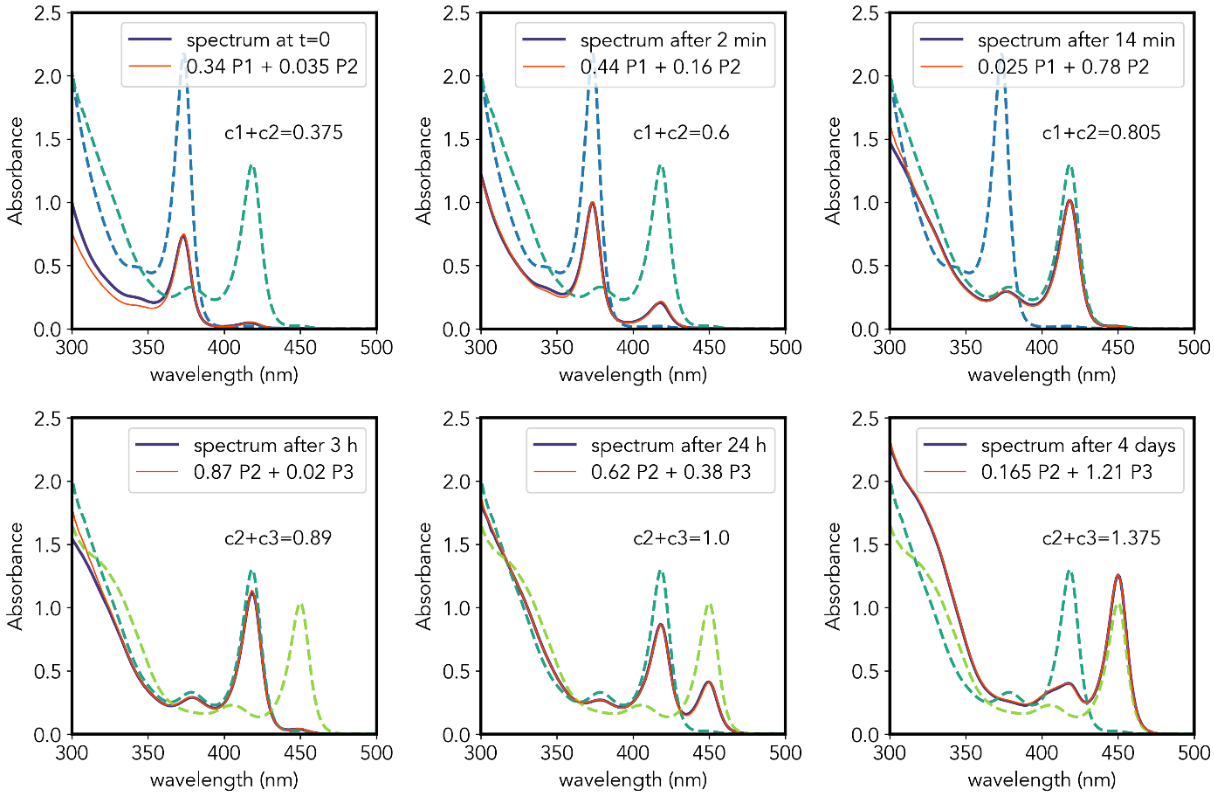

Figure S17. Temporal evolution of the absorption spectra of the CdTe products obtained for **[DDA] = 0.58 M**. Each panel corresponds to one time point, indicated in the legend. The measured spectra are given in blue. The first measurement (indicated by "0") was carried out ~30 s after the preparation of the reaction mixture. The linear combinations (LC) of the spectra of the three single-species MSNWs (NW-373, NW-418, and NW-450, spectra P1, P2, and P3, in Figure S15c above) that best reproduce the experimental spectra are plotted in orange. The single-species spectra used in each LC are plotted as dashed lines, using the same color code used in Figure S15c. The legend also gives the equation describing the best LC and the sum of the relevant coefficients c*N* (*N*= 1, 2, or 3) at each time point.

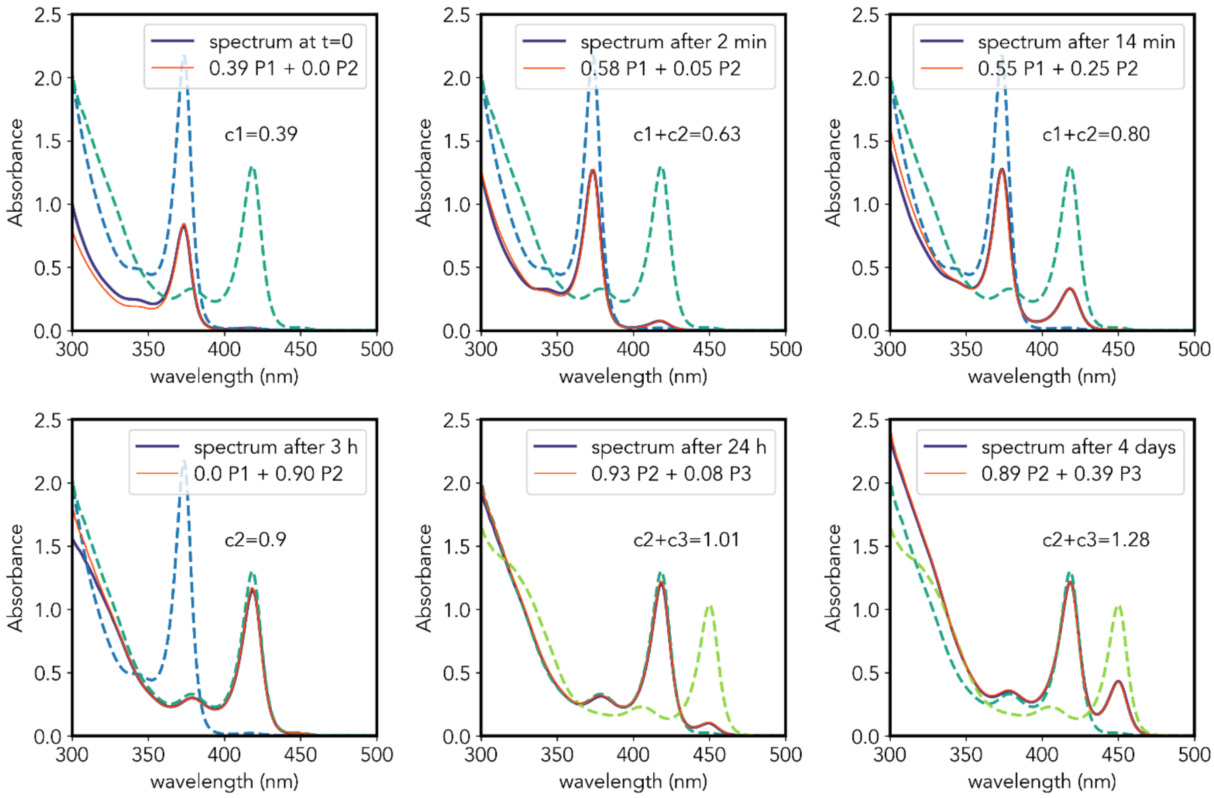

Figure S18. Temporal evolution of the absorption spectra of the CdTe products obtained for **[DDA] = 0.73 M**. Each panel corresponds to one time point, indicated in the legend. The measured spectra are given in blue. The first measurement (indicated by "0") was carried out ~30 s after the preparation of the reaction mixture. The linear combinations (LC) of the spectra of the three single-species MSNWs (NW-373, NW-418, and NW-450, spectra P1, P2, and P3, in Figure S15c above) that best reproduce the experimental spectra are plotted in orange. The single-species spectra used in each LC are plotted as dashed lines, using the same color code used in Figure S15c. The legend also gives the equation describing the best LC and the sum of the relevant coefficients c*N* (*N*= 1, 2, or 3) at each time point.

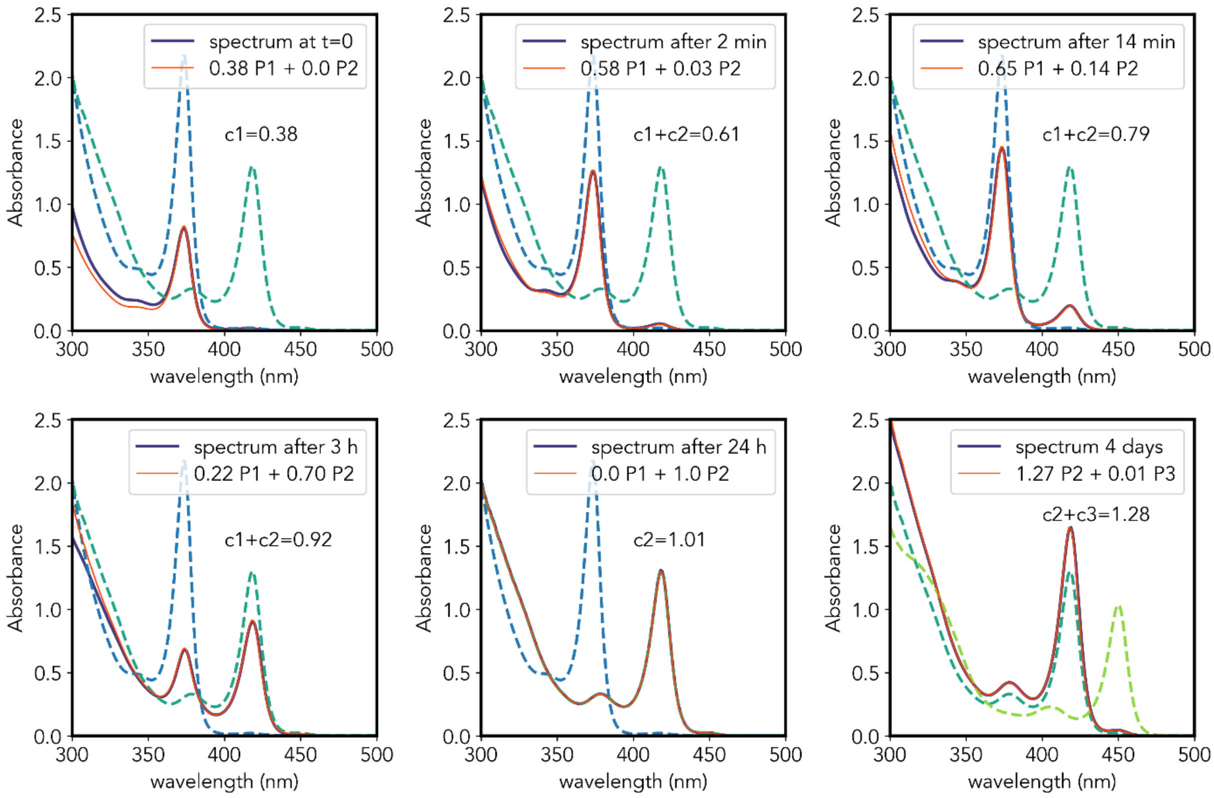

Figure S19. Temporal evolution of the absorption spectra of the CdTe products obtained for **[DDA] = 0.87 M**. Each panel corresponds to one time point, indicated in the legend. The measured spectra are given in blue. The first measurement (indicated by "0") was carried out ~30 s after the preparation of the reaction mixture. The linear combinations (LC) of the spectra of the three single-species MSNWs (NW-373, NW-418, and NW-450, spectra P1, P2, and P3, in Figure S15c above) that best reproduce the experimental spectra are plotted in orange. The single-species spectra used in each LC are plotted as dashed lines, using the same color code used in Figure S15c. The legend also gives the equation describing the best LC and the sum of the relevant coefficients c*N* (*N*= 1, 2, or 3) at each time point.

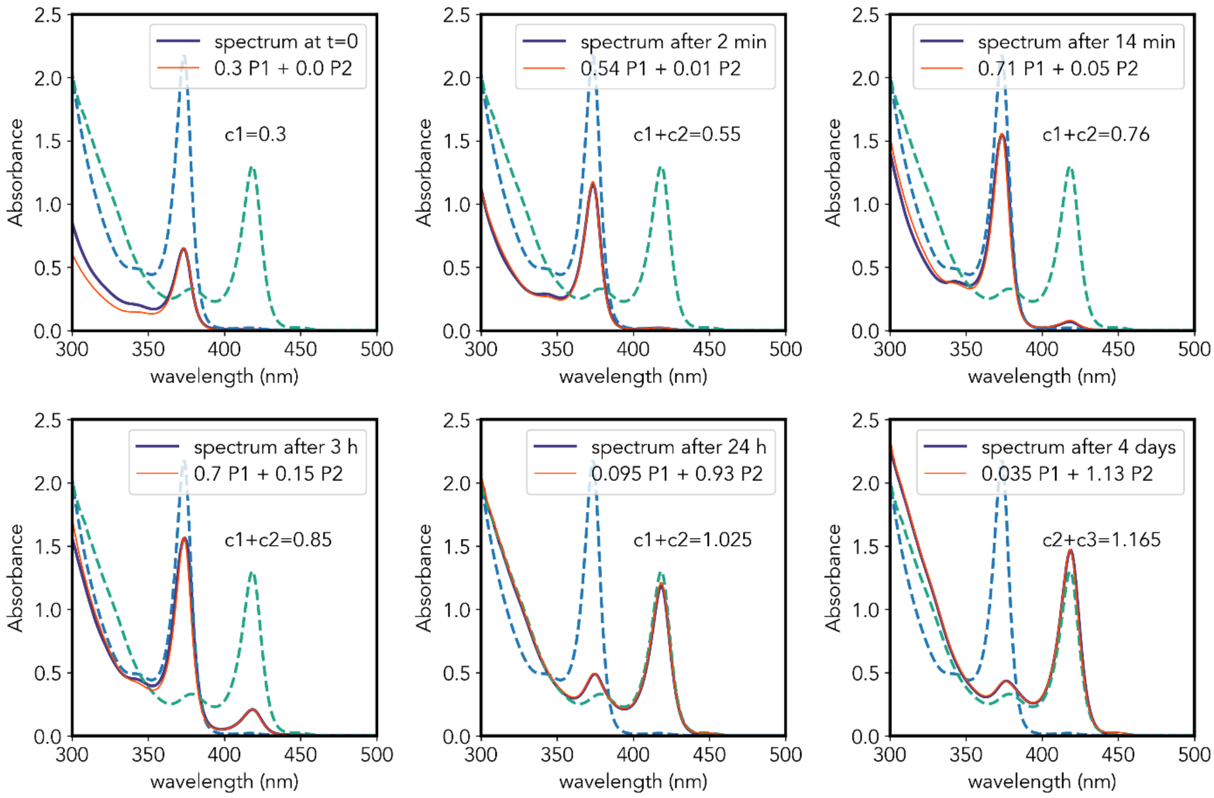

Figure S20. Temporal evolution of the absorption spectra of the CdTe products obtained for **[DDA] = 1.17 M**. Each panel corresponds to one time point, indicated in the legend. The measured spectra are given in blue. The first measurement (indicated by “0”) was carried out ~30 s after the preparation of the reaction mixture. The linear combinations (LC) of the spectra of the three single-species MSNWs (NW-373, NW-418, and NW-450, spectra P1, P2, and P3, in Figure S15c above) that best reproduce the experimental spectra are plotted in orange. The single-species spectra used in each LC are plotted as dashed lines, using the same color code used in Figure S15c. The legend also gives the equation describing the best LC and the sum of the relevant coefficients c*N* (*N*= 1, 2, or 3) at each time point.

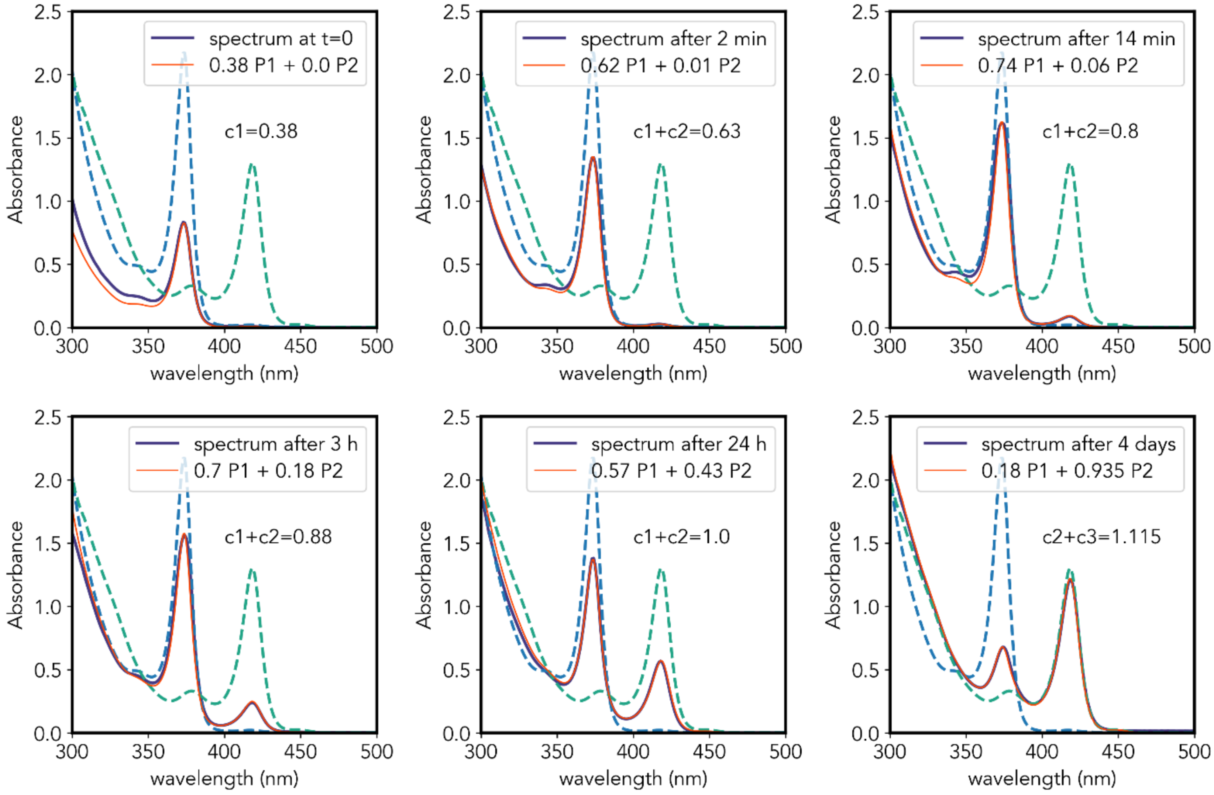

Figure S21. Temporal evolution of the absorption spectra of the CdTe products obtained for **[DDA] = 1.46 M**. Each panel corresponds to one time point, indicated in the legend. The measured spectra are given in blue. The first measurement (indicated by "0") was carried out ~30 s after the preparation of the reaction mixture. The linear combinations (LC) of the spectra of the three single-species MSNWs (NW-373, NW-418, and NW-450, spectra P1, P2, and P3, in Figure S15c above) that best reproduce the experimental spectra are plotted in orange. The single-species spectra used in each LC are plotted as dashed lines, using the same color code used in Figure S15c. The legend also gives the equation describing the best LC and the sum of the relevant coefficients c*N* (*N*= 1, 2, or 3) at each time point.

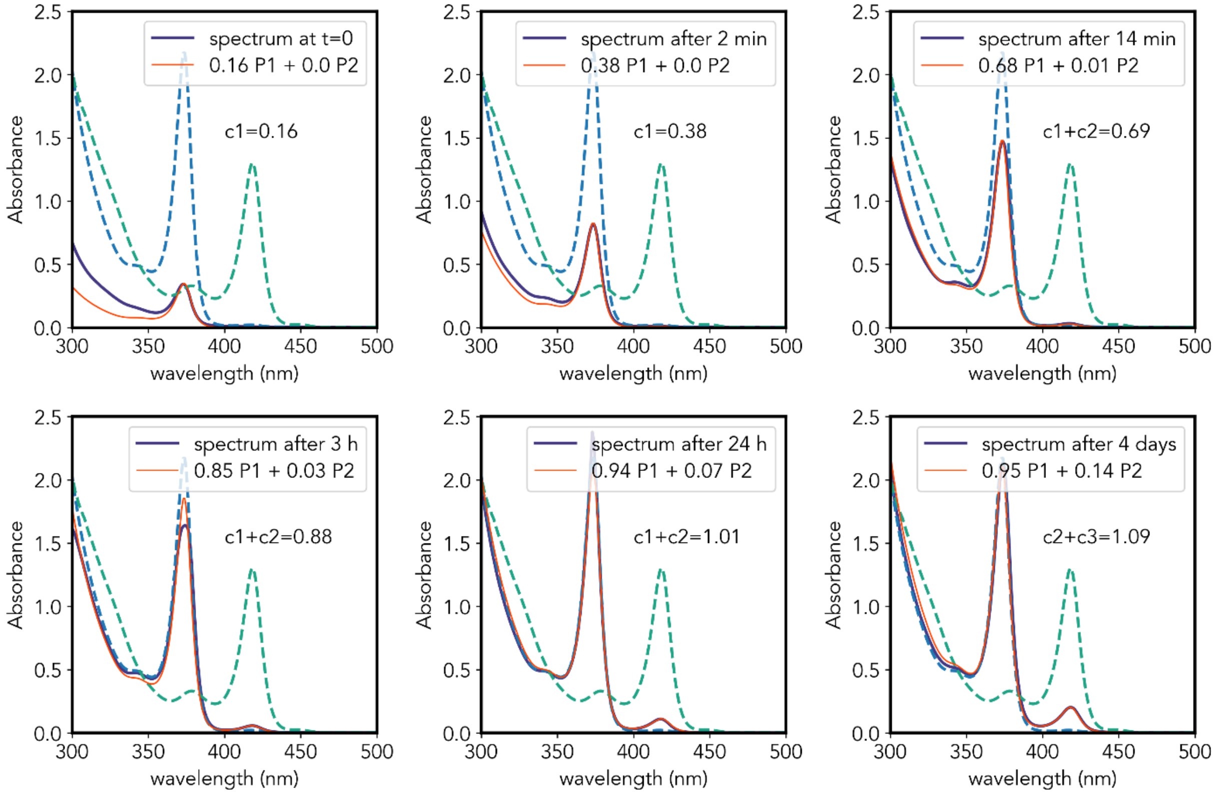

Figure S22. Temporal evolution of the absorption spectra of the CdTe products obtained for **[DDA] = 1.75 M**. Each panel corresponds to one time point, indicated in the legend. The measured spectra are given in blue. The first measurement (indicated by "0") was carried out ~30 s after the preparation of the reaction mixture. The linear combinations (LC) of the spectra of the three single-species MSNWs (NW-373, NW-418, and NW-450, spectra P1, P2, and P3, in Figure S15c above) that best reproduce the experimental spectra are plotted in orange. The single-species spectra used in each LC are plotted as dashed lines, using the same color code used in Figure S15c. The legend also gives the equation describing the best LC and the sum of the relevant coefficients c*N* (*N*= 1, 2, or 3) at each time point.

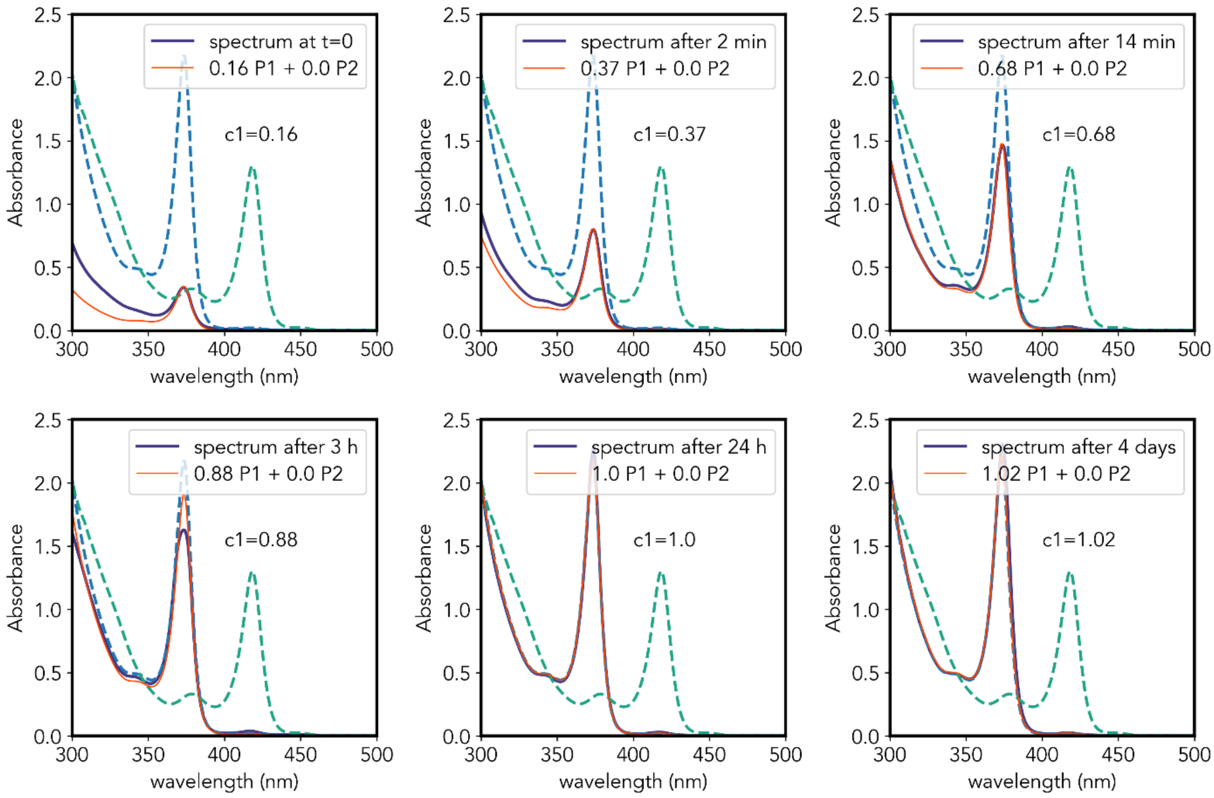

Figure S23. Temporal evolution of the absorption spectra of the CdTe products obtained for **[DDA] = 2.04 M**. Each panel corresponds to one time point, indicated in the legend. The measured spectra are given in blue. The first measurement (indicated by "0") was carried out ~30 s after the preparation of the reaction mixture. The linear combinations (LC) of the spectra of the three single-species MSNWs (NW-373, NW-418, and NW-450, spectra P1, P2, and P3, in Figure S15c above) that best reproduce the experimental spectra are plotted in orange. The single-species spectra used in each LC are plotted as dashed lines, using the same color code used in Figure S15c. The legend also gives the equation describing the best LC and the sum of the relevant coefficients c*N* (*N*= 1, 2, or 3) at each time point.

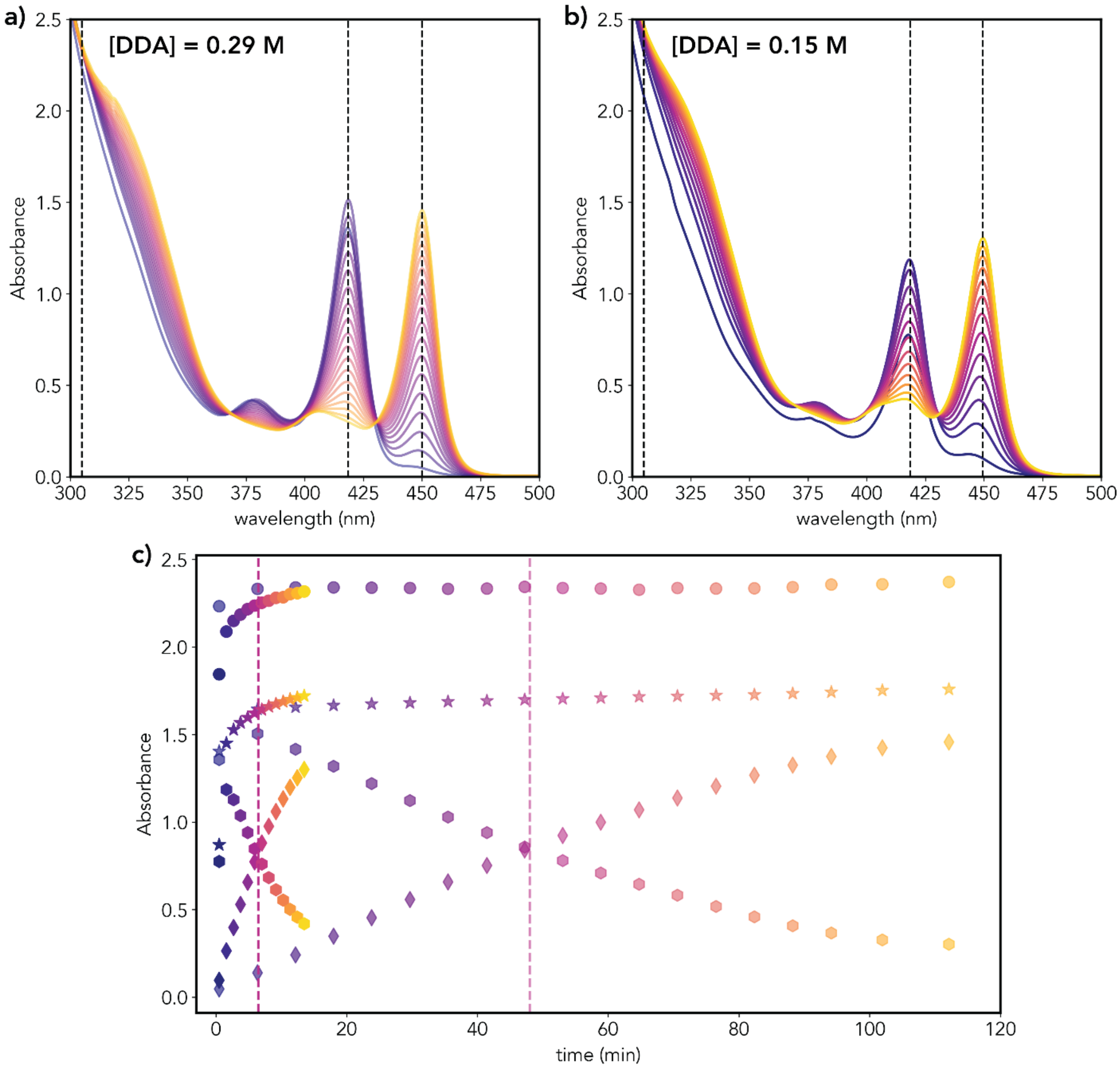

Figure S24. **(a, b)** Temporal evolution of the absorption spectra of the CdTe products obtained for two different DDA concentrations, acquired in quartz cuvettes with 1-mm optical path using the neat reaction mixture. All other reaction variables are the same. The time intervals between two successive spectra are 6.5 min in **(a)** and 1.5 min in **(b)**. **(c)** Temporal evolution of the absorbance at 300 nm (circles), at 417 nm (hexagons), and at 450 nm (diamonds) and of the sum of the absorbance at 417 and 450 nm (stars), for the spectra shown in panel **(a)** (symbols in faded colors) and in panel **(b)** (symbols in bright colors). The vertical dashed lines mark the times at which the absorbances at 417 and 450 nm are equal (*i.e.*, the isosbestic points).

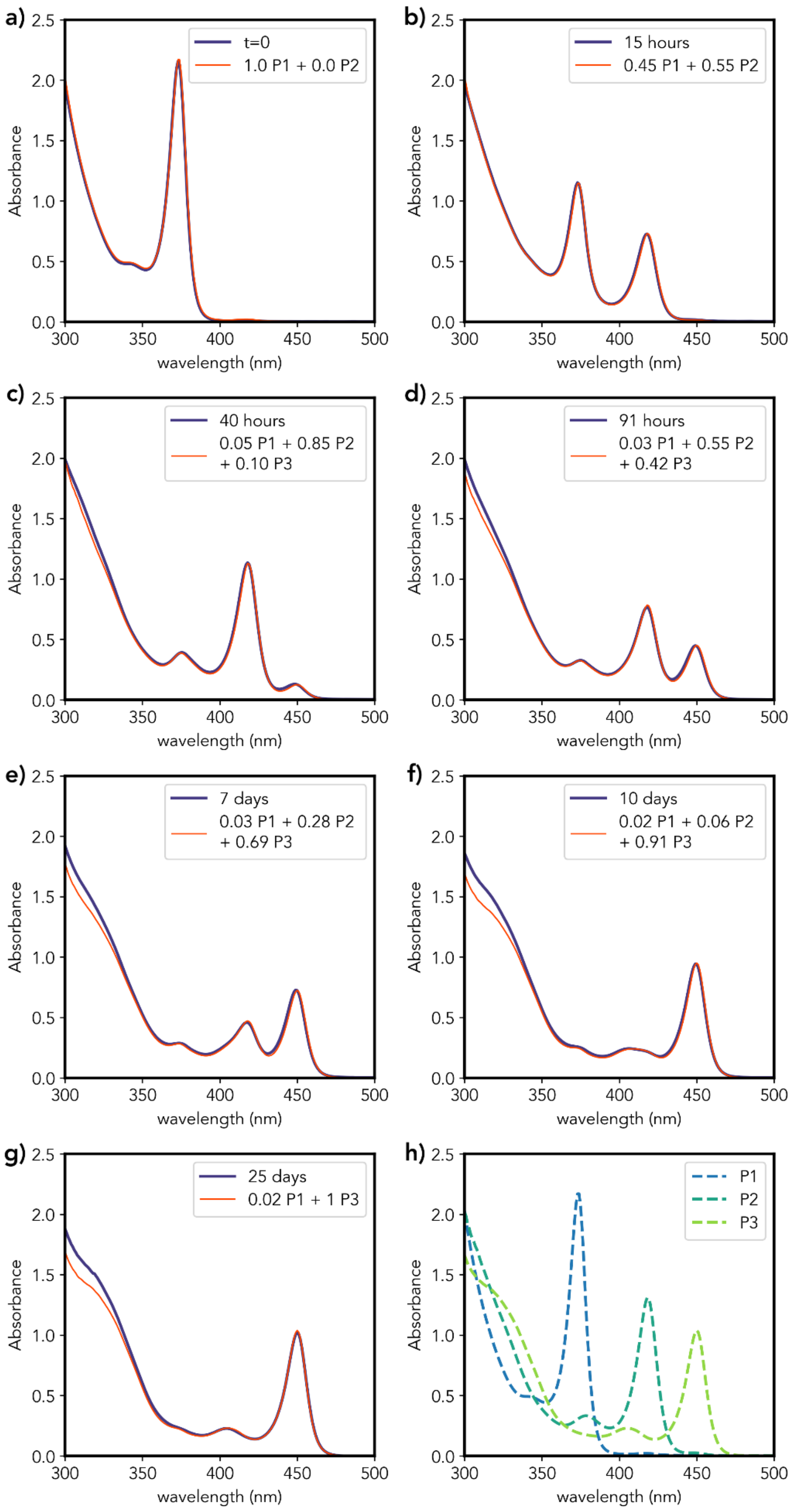
a)
t=0
1.0 P1 + 0.0 P2
b)
15 hours
0.45 P1 + 0.55 P2
c)
40 hours
0.05 P1 + 0.85 P2 + 0.10 P3
d)
91 hours
0.03 P1 + 0.55 P2 + 0.42 P3
e)
7 days
0.03 P1 + 0.28 P2 + 0.69 P3
f)
10 days
0.02 P1 + 0.06 P2 + 0.91 P3
g)
25 days
0.02 P1 + 1 P3
h)
P1
P2
P3
Absorbance
wavelength (nm)

Figure S25. **(a-g)**. Temporal evolution of the absorption spectra of a solution initially containing single-species CdTe NW-373 ([DDA]= 2.04 M in the reaction mixture), after its dilution to a DDA concentration that stabilizes CdTe NW-450 ([DDA]= 0.29 M). The measurements were carried out in a sealed 10-mm quartz cuvette. The first measurement (0 min) was acquired immediately after the dilution. The experimental spectra (blue solid lines) are reproduced by linear combinations (solid orange lines) of the spectra of the three single-species MSNWs (NW-373, NW-418, and NW-450, spectra P1, P2, and P3, in Figure S15c above). The legend also gives the equation describing the best linear combination at each time point. **(h)** The single-species spectra used in the linear combinations. The color code is the same used in Figures S15-S23.

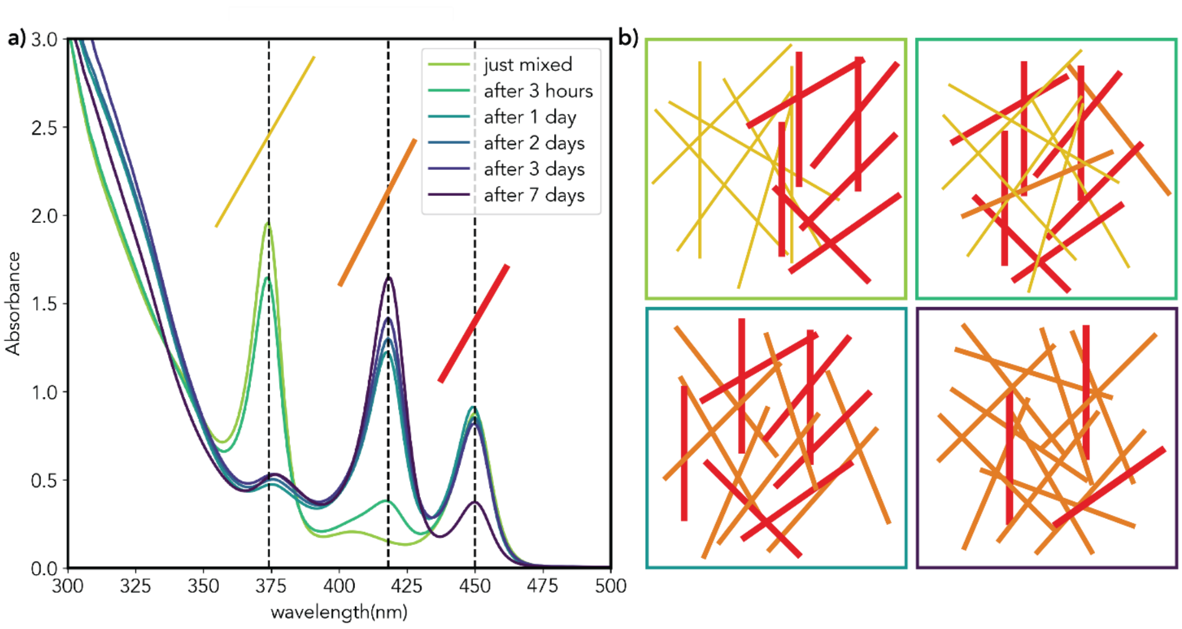

Figure S26. **(a)** Temporal evolution of the absorption spectra of a mixture of equal volumes of solutions initially containing single-species CdTe NW-450 ($[DDA]_0$= 0.29 M) and single-species CdTe NW-373 ($[DDA]_0$= 2.04 M). Prior to the mixing both solutions had been allowed to react for 4 days to ensure that the reaction had reached completion (see Figures S16 and S23 above). The final DDA concentration ($[DDA]_{new}$) is 1.16 M, which stabilizes single-species CdTe NW-418 (see Figure S20 above). After 7 days most NW-450 and NW-373 had been converted to NW-418. **(b)** Cartoon schematically showing the evolution of the system after the mixture of the two single-species MSNWs. The three different single-species MSNWs are represented by yellow (thinnest MSNWs, 373-NW, 1st absorption peak at 373 nm, 0.7 nm diameter), orange (intermediate MSNWs, 418-NW, 1st absorption peak at 418 nm, 0.9 nm diameter), and red (thickest MSNWs, 450-NW, 1st absorption peak at 450 nm, 1.1 nm diameter) sticks. The system is shown at four time points after the mixing, using the same color code as in panel (a) for the frames.
